# Corticosteroid treatment and mortality in mechanically ventilated COVID-19-associated acute respiratory distress syndrome (ARDS) patients: a multicentre cohort study

**DOI:** 10.1186/s13613-021-00951-0

**Published:** 2021-11-26

**Authors:** Gerard Moreno, Raquel Carbonell, Ignacio Martin-Loeches, Jordi Solé-Violán, Eudald Correig i Fraga, Josep Gómez, Manuel Ruiz-Botella, Sandra Trefler, María Bodí, Josefa Murcia Paya, Emili Díaz, Pablo Vidal-Cortes, Elisabeth Papiol, Antonio Albaya Moreno, Susana Sancho Chinesta, Lorenzo Socias Crespi, María del Carmen Lorente, Ana Loza Vázquez, Rebeca Vara Arlanzon, María Teresa Recio, Juan Carlos Ballesteros, Ricard Ferrer, Elisabeth Fernandez Rey, Marcos I. Restrepo, Ángel Estella, Antonio Margarit Ribas, Neus Guasch, Luis F. Reyes, Judith Marín-Corral, Alejandro Rodríguez, the COVID-19 SEMICYUC Working Group

**Affiliations:** 1 Critical Care Department, Autonomous University of Barcelona (UAB), Joan XXIII University Hospital, C/ Dr Mallafrè Guasch, 4, 43005 Tarragona, Spain; 2 Department of Intensive Care Medicine, Multidisciplinary Intensive Care Research Organization (MICRO), St. James’s Hospital, Dublin, Ireland; 3 Critical Care Department, Doctor Negrín University Hospital, Gran Canaria, Spain; 4 Department of Biostatistics, University of Rovira i Virgili (URV), Reus, Spain; 5 Tarragona Health Data Research Working Group (THeDaR), Joan XXIII University Hospital, Tarragona, Spain; 6 Critical Care Department, URV/IISPV/CIBERES, Joan XXIII University Hospital, Tarragona, Spain; 7 Critical Care Department, Santa Lucía General University Hospital, Cartagena, Spain; 8 Critical Care Department, Autonomous University of Barcelona (UAB), Parc Taulí Hospital, Sabadell, Spain; 9 Critical Care Department, Ourense University Hospital, Ourense, Spain; 10 Critical Care Department, Vall d’Hebrón University Hospital, Barcelona, Spain; 11 Critical Care Department, UAH, Guadalajara University Hospital, Guadalajara, Spain; 12 Critical Care Department, University and Polytechnic Hospital of La Fe, Valencia, Spain; 13 Critical Care Department, Son Llàtzer Hospital, Palma de Mallorca, Spain; 14 Critical Care Department, Rafael Méndez Hospital, Murcia, Spain; 15 Critical Care Department, Virgen de Valme University Hospital, Sevilla, Spain; 16 Critical Care Department, Burgos University Hospital, Burgos, Spain; 17 Critical Care Department, University Hospital of Salamanca, Salamanca, Spain; 18 Critical Care Department, Investigation Group SODIR-VIHR, Vall d’Hebrón University Hospital, Barcelona, Spain; 19 Critical Care Department, University Central Hospital of Asturias, Oviedo, Spain; 20 Department of Medicine, South Texas Veterans Health Care System and University of Texas Health, San Antonio, TX USA; 21 Critical Care Department, Jerez University Hospital, Jerez, Spain; 22 Critical Care Department, Nostra Senyora de Meritxell Hospital, Escaldes-Engordany, Andorra; 23 Infectious Diseases Department, Universidad de La Sabana, Chía, Colombia; 24 Autonomous University of Barcelona (UAB) – Institut Hospital del Mar d’Investigacions Mèdiques (IMIM), Barcelona, Spain

**Keywords:** Corticosteroids, COVID-19-associated acute respiratory distress syndrome, Intensive care unit, Mortality, Invasive mechanical ventilation

## Abstract

**Background:**

Some unanswered questions persist regarding the effectiveness of corticosteroids for severe coronavirus disease 2019 (COVID-19) patients. We aimed to assess the clinical effect of corticosteroids on intensive care unit (ICU) mortality among mechanically ventilated COVID-19-associated acute respiratory distress syndrome (ARDS) patients.

**Methods:**

This was a retrospective study of prospectively collected data conducted in 70 ICUs (68 Spanish, one Andorran, one Irish), including mechanically ventilated COVID-19-associated ARDS patients admitted between February 6 and September 20, 2020. Individuals who received corticosteroids for refractory shock were excluded. Patients exposed to corticosteroids at admission were matched with patients without corticosteroids through propensity score matching. Primary outcome was all-cause ICU mortality. Secondary outcomes were to compare in-hospital mortality, ventilator-free days at 28 days, respiratory superinfection and length of stay between patients with corticosteroids and those without corticosteroids. We performed survival analysis accounting for competing risks and subgroup sensitivity analysis.

**Results:**

We included 1835 mechanically ventilated COVID-19-associated ARDS, of whom 1117 (60.9%) received corticosteroids. After propensity score matching, ICU mortality did not differ between patients treated with corticosteroids and untreated patients (33.8% vs. 30.9%; *p* = 0.28). In survival analysis, corticosteroid treatment at ICU admission was associated with short-term survival benefit (HR 0.53; 95% CI 0.39–0.72), although beyond the 17th day of admission, this effect switched and there was an increased ICU mortality (long-term HR 1.68; 95% CI 1.16–2.45). The sensitivity analysis reinforced the results. Subgroups of age < 60 years, severe ARDS and corticosteroids plus tocilizumab could have greatest benefit from corticosteroids as short-term decreased ICU mortality without long-term negative effects were observed. Larger length of stay was observed with corticosteroids among non-survivors both in the ICU and in hospital. There were no significant differences for the remaining secondary outcomes.

**Conclusions:**

Our results suggest that corticosteroid treatment for mechanically ventilated COVID-19-associated ARDS had a biphasic time-dependent effect on ICU mortality. Specific subgroups showed clear effect on improving survival with corticosteroid use. Therefore, further research is required to identify treatment-responsive subgroups among the mechanically ventilated COVID-19-associated ARDS patients.

**Supplementary Information:**

The online version contains supplementary material available at 10.1186/s13613-021-00951-0.

## Background

It has been more than a year since the devastating severe acute respiratory syndrome coronavirus 2 (SARS-CoV-2) outbreak occurred, which emerged in Wuhan (China) on December 2019, and the global health issue is still a concern to resolve. As of July 2021, the World Health Organization reported more than 180 million cases and four million deaths worldwide due to coronavirus disease 2019 (COVID-19) [1]. The clinical presentation ranges from asymptomatic or mild-to-severe pneumonia in which the most critical cases develop life-threatening acute respiratory distress syndrome (ARDS), requiring admission to the intensive care unit (ICU) with high rates of invasive mechanical ventilation and mortality [2].

Worldwide, corticosteroids have become the standard of care (SOC) for severe COVID-19 patients, especially in mechanically ventilated patients since the results of the RECOVERY trial [3] and the subsequent meta-analysis [4] from seven randomized clinical trials (RCTs) revealed a reduction in 28-day mortality. However, several questions remain unanswered regarding the effectiveness of corticosteroids [5, 6]. It is unclear if there are particular subsets of COVID-19 patients under mechanical ventilation with different severity of illness or ARDS degree in whom corticosteroids perhaps had less pronounced effect and caution in the use of corticosteroids should be exercised. Clinical effectiveness of corticosteroid treatment in COVID-19 patients with ARDS is still limited and conflicted by the results from some meta-analysis [7, 8] and observational data [9–11].

We hypothesize that corticosteroid use may have to be individualized for corticosteroid-responsiveness COVID-19-associated ARDS patients, recognizing patient heterogeneity according to patient characteristics, severity of the disease, timing of the illness and other complications that occur during mechanical ventilation. Therefore, the aim of this study was to determine the association of corticosteroid treatment and ICU mortality among mechanically ventilated COVID-19-associated ARDS patients.

## Methods

### Study design and participants

This was a retrospective analysis of prospectively collected data of consecutive subjects admitted to 70 ICUs (68 Spanish, one Andorran, and one Irish) from February 6 to September 20, 2020. Data were collected through a registry created by the Spanish Society of Intensive Care Medicine and Coronary Units (SEMICYUC). Data were recorded using a case report form which included all information gathered from medical records, radiologic findings, and laboratory results during ICU admission. All data were de-identified, allowing the waiver of informed consent. The study was approved by the Ethics Committee board of Joan XXIII University Hospital (IRB# CEIM/066/2020). The study was registered in ClincalTrials.gov (NCT04948242) and followed the Strobe guidelines (see Additional file 1: Table S1). The end of follow-up was completed when patients were discharged alive or deceased in ICU.

Consecutive patients older than 16 years of age were eligible for participation if admitted to one of the participating ICUs and had received invasive mechanical ventilation for COVID-19 within the first day of ICU admission (Additional file 1: Fig. S1). A COVID-19 diagnosis had to be confirmed by a positive reverse transcriptase-polymerase chain reaction for SARS-CoV-2 from upper or lower respiratory tract samples [12]. The diagnosis of ARDS was fulfilled if patients had acute timing of respiratory failure within a week due to viral pneumonia and bilateral opacities on chest imaging not fully explained by cardiac failure or overload according the Berlin definition [13]. The severity of ARDS was classified as mild, moderate and severe accordingly with partial pressure of oxygen in arterial blood to fraction of inspired oxygen (PaO_2_/FiO_2_) ratio of < 300 to 200, < 200 to 100, and < 100 mmHg with levels of positive end-expiratory pressure (PEEP) ≥ 5 cmH_2_O, respectively.

For the current analysis, we excluded patients with: (1) ICU length of stay less than 2 days, and (2) corticosteroid use for confounding factors (patients with corticosteroids for refractory shock).

### Data collection

Extracted data included demographic characteristics, comorbidities, time course of illness, treatments, laboratory, microbiologic and radiological findings, ventilatory parameters on day one of ICU admission, complications during ICU stay, and outcomes (Additional file 1: Table S2). Type of corticosteroid and duration of treatment were also recorded. Illness severity was determined with the Acute Physiology and Chronic Health Evaluation (APACHE) II score calculated at 24 h of ICU admission and organ failure was assessed using the Sequential Organ Failure Assessment (SOFA) score at ICU admission. Ventilatory management strategies were not standardized between centres and were left to the discretion of the attending clinician and based on SEMICYUC and National Ministry of Health recommendations, supported by international guidelines [14].

Corticosteroid treatment was defined as the administration of systemic corticosteroids within the first day of ICU admission, prescribed as adjuvant treatment for pneumonia. Patients who started corticosteroids within the 2 days before ICU admission were also considered exposed. Most patients were admitted during the first wave of the pandemic when corticosteroids were not SOC yet for severe COVID-19 patients, reason that explained why almost 40% of patients did not receive corticosteroids (Additional file 1: Fig. S2). Therefore, the decision to prescribe corticosteroids at ICU admission was based on the criteria of the attending physician based on clinical markers as well as arterial blood gas measurements. Ventilator-associated pneumonia (VAP) was diagnosed as the microbiologic confirmed pneumonia developed in ICU patients who have been mechanically ventilated for at least 48 h [15]. The remaining definitions are summarized in Additional file 1.

### Outcomes

The primary outcome was all-cause ICU mortality. Secondary outcomes were in-hospital mortality, ventilator-free days at 28 days, respiratory superinfection, and length of stay (LOS) between patients with corticosteroids compared with those without corticosteroids.

### Statistical analysis

No statistical sample size calculation was performed and sample size was equal to the number of patients admitted to the participating ICUs during the study period. Discrete variables were expressed as counts (percentages), while continuous variables were expressed as medians with interquartile ranges (IQR). For baselines characteristics, differences between groups were assessed using Chi-squared and Fisher’s exact tests for categorical variables or the Mann–Whitney *U* test for continuous variables. Significant differences were considered if *p* values were < 0.05 for a two-tailed test. Missing data were handled with multiple imputation by chained equations (Additional file 1: Table S3). All patients in the corticosteroid group initiated the treatment within the first day of ICU stay at the latest. Time zero of follow-up was ICU admission, but we discarded all patients censored within 48 h of ICU admission to avoid immortal time bias. Propensity score through Genetic matching algorithm was used to reduce treatment selection bias and balance the covariance matrix for both groups [16]. The matching was 1:1 with replacement and ties, and without calipers. Variables selected for the inclusion into the matching model were those baseline variables related to the outcome [17]. Those variables covered demographic characteristics and comorbidities, disease severity, laboratory data, organ failures and potential treatments affecting the survival (tocilizumab). Logistic regression analysis was performed to investigate the association of corticosteroids and ICU mortality in the matched cohort. These results were presented as odds ratios (OR) and 95% confidence intervals (CI). As a complementary analysis to the main outcome, we conducted a survival analysis to investigate whether survival times were related to covariates, and estimating the effect size of a corticosteroid treatment after adjusting for potential confounders. Owing to the fact that being discharged alive has been identified as a competitive event for ICU mortality [18], the survival analysis with Cox regression was performed using a competing risks analysis through a cause-specific hazard model [19, 20]. A competing risk (discharged alive) is an event whose occurrence precludes the occurrence of the primary event of interest (ICU mortality). In survival analysis in which competing risks are present, censoring patients who have been discharge alive may be misleading as it may violate the assumption of non-informative censoring. Thus, for etiological research the proportional cause-specific hazard model is a suitable method in case of competing risks analysis. In such case, the Cox regression analysis is applied for each of the specific types of events (both ICU mortality and discharged alive) and it directly quantify the estimates hazard ratios among subjects who are actually at risk of developing the event of interest.

When the Cox model was accomplished, the proportional hazards assumption was strongly violated for corticosteroids (Additional file 1: Fig. S3). Proportional hazards may not hold over the entire time axis, but may hold approximately over shorter time periods. The effect of a time-varying covariate (corticosteroid treatment) becomes stronger or weaker over time, which can be explored via stratification by time. Therefore, we carried out a time-dependent Cox regression using a step function to deal with non-proportional hazards. The step function consisted in a partitioning of the time axis dividing the follow-up into shorter time periods, hence the proportional hazard assumption was met within each interval of the partition [21]. We established to divide the study time frame in two intervals (at 17th of follow-up) when the proportional hazards assumption was met. The rationale behind the 17-day-step term was based on the pronounced change of case-fatality rates according with both the life-tables and the survival curves in this timeframe. With this methodology, we modelled the effect of corticosteroids on mortality in two ranges: the short and long-term. The results of time-to-event data were expressed as hazard ratios (HR) and 95% CI.

Prespecified subgroup sensitivity analysis with exploratory nature was performed with propensity score matching for each study subgroup to evaluate whether the observed effect of corticosteroids on ICU mortality was consistent across subgroups, and to assess the robustness of our findings. ICU mortality was investigated either by comparing proportions in the matched subsets and survival analysis with cause-specific hazard model. Subgroups were based on previous research as well as clinical relevance and categorized as: age (< 60, ≥ 60), severity of ARDS (mild, moderate and severe), and time since the symptom onset to the initiation of corticosteroids (< 7 days, ≥ 7 days). We also evaluated the subgroups of corticosteroid treatment duration (< 7 days, ≥ 7 days) and tocilizumab (yes, no) as post hoc analysis. To account for multiplicity and avoid the potential inflation of the type I error rate as a result of multiple testing in the subgroup analysis, we used the Benjamini–Hochberg method for controlling the false discovery rate.

Ventilator-free days at 28 days were calculated as the number of days alive and free from mechanical ventilation for at least 48 h without reintubation (successful liberation) in patients who have survived 28 days after ICU admission, and for patients ventilated 28 days or more, or who died before 28 days (irrespective of ventilated status), the number of ventilator-free days was recorded at zero. Results are expressed as means and standard deviation (SD) and with a competing risks analysis using the Fine and Gray model [22].

Centre effect for ICU mortality was investigated by multilevel logistic regression analysis through a conditional random intercept model using inter-hospital variation as a random-effects variable. Regression coefficients were summarized as the variance with standard deviation (SD) and the interclass correlation coefficient (ICC). Data analysis were done with SPSS version 24 (IBM Corp. Armonk, NY, USA) and R v.3.6 (cran.r-project.org).

## Results

**Fig. 1:**
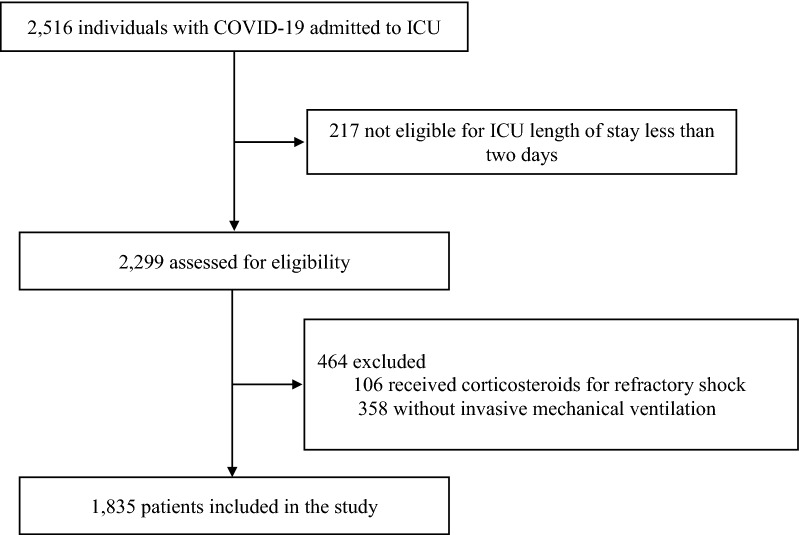
Study flowchart

Between February 6 and September 20, 2020, data from 2516 critically ill COVID-19 patients were collected. We included 1835 mechanically ventilated COVID-19-associated ARDS patients in this analysis, of whom 1117 (60.9%) received corticosteroid treatment (Fig. 1).

### Demographics and characteristics

The median age was 65 years (IQR 56–72) and 1290 (70.3%) were male. The median baseline APACHE II and SOFA scores were 15 (IQR 11–19) and 6 (IQR 4–8), respectively. The median time from symptom onset to COVID-19 diagnosis was 6 (IQR 4–8) days and the time from hospital to ICU admission was 2 (IQR 0–4) days. Almost three out of four (*n* = 1361; 74.2%) patients had at least one comorbidity. The most frequent underlying condition was hypertension (*n* = 852; 46.4%). All patients were intubated for acute respiratory failure, after the failure of conventional oxygen therapy (*n* = 278, 15.1%), high-flow nasal cannula (*n* = 260; 14.2%), or non-invasive mechanical ventilation (*n* = 64; 3.5%), while the remaining (*n* = 1234; 67.2%) needed invasive mechanical ventilation as the first line respiratory support due to promptly failure of the initial oxygen therapy. Within the first day of ICU admission, 476 (25.9%) were classified as mild, 933 (50.8%) as moderate, and 426 (23.2%) as severe ARDS, respectively (Additional file 1: Table S4). Patients were profoundly hypoxaemic with a median value of PaO_2_/FiO_2_ of 141 (IQR 98–188) mmHg and prone position was frequently used (*n* = 1186; 64.6%). Lung-protective ventilation was widely implemented, with tidal volume of 6.5 (IQR 6–7) ml/kg/predicted body weight, moderate levels of PEEP (median 12 [IQR 10–14] cmH_2_O), and targeted plateau pressures (median 25 [IQR 22–29] cmH_2_O).

After propensity score matching, 1117 patients with corticosteroid treatment and 463 patients without corticosteroids were matched (Table 1). Several covariates were well balanced between groups and the summaries of balanced data of variables included into the matching model are shown in Additional file 1: Fig. S4. The matched cohort was appropriately well-adjusted in terms of demographic characteristics, illness severity, comorbidities (such as cardiovascular risk factors and immunosuppression status), time course of the disease, laboratory data, complications at admission (such as organ failures, myocardial dysfunction and co-infection) as well as in co-adjuvant treatments.

**Table 1:** Baseline characteristics of groups according to corticosteroid treatment before and after propensity score matching

	Unmatched cohort (*n* = 1835)				Matched cohort (*n* = 1580)			
	Corticosteroids (*n* = 1117)	No corticosteroids (*n* = 718)	Smd	*P* value	Corticosteroids (*n* = 1117)	No corticosteroids (*n* = 463)	Smd	*P* value
Demographic characteristics								
Age (years)	64 [57–71]	65 [55–72]	− 0.03	0.31	64 [57–71]	66 [57–72]	− 0.06	0.33
Gender			0.04	0.44			0.05	0.38
Male	793 (71%)	497 (69.2%)			793 (71%)	339 (73.2%)		
Female	324 (29%)	221 (30.8%)			324 (29%)	124 (26.8%)		
BMI (kg/m^2^)	28 [26–31]	28 [26–31]	0.02	0.45	28 [26–31]	28 [26–31]	0.03	0.82
Time course of disease (days)								
Diagnosis gap^a^	6 [4–8]	6 [4–8]		0.78	6.2 [4–8]	6.5 [5–8]		0.33
Hospital gap^b^	7 [5–8]	6 [4–8]	0.06	0.09	7 [5–8]	6.2 [5–8]	0.05	0.37
ICU gap^c^	2 [0–4]	1 [0–3]	0.06	0.02	2 [0–4]	1 [0–3]	0.14	0.11
Comorbidities								
Any comorbidity	825 (73.9%)	536 (74.6%)	− 0.02	0.74	825 (73.9%)	339 (73.2%)	0.01	0.84
Hypertension	512 (45.8%)	340 (47.3%)	− 0.03	0.55	512 (45.8%)	213 (46%)	− 0.01	0.99
Prior ACE inhibitors	173 (15.5%)	128 (17.8%)		0.20	173 (15.5%)	87 (18.8%)		0.12
Prior ARBs	201 (18%)	126 (17.5%)		0.85	201 (18%)	68 (14.8%)		0.13
Diabetes mellitus	236 (21.1%)	143 (20%)	0.03	0.57	236 (21.1%)	85 (18.4%)	0.06	0.25
Dyslipidaemia	120 (10.7%)	68 (9.5%)	0.04	0.42	120 (10.7%)	40 (8.7%)	0.06	0.27
Ischaemic heart disease	73 (6.5%)	49 (6.8%)	− 0.01	0.88	73 (6.5%)	25 (5.5%)	0.04	0.49
Asthma	80 (7.2%)	41 (5.7%)	0.06	0.25	80 (7.2%)	28 (6%)	0.04	0.47
COPD	79 (7.1%)	61 (8.5%)	− 0.05	0.30	79 (7.1%)	25 (5.4%)	0.06	0.77
Chronic kidney disease	46 (4.1%)	31 (4.3%)	− 0.01	0.92	46 (4.1%)	15 (3.2%)	0.04	0.48
Immunosuppression	40 (3.6%)	24 (3.3%)	0.01	0.88	40 (3.6%)	15 (3.3%)	0.01	0.90
Haematological disease	34 (3%)	33 (4.6%)	− 0.09	0.10	34 (3%)	14 (3%)	0.01	0.99
Autoimmune disease	47 (4.2%)	30 (4.2%)	0.01	0.99	47 (4.2%)	17 (3.7%)	0.02	0.78
Neuromuscular disease	9 (0.8%)	9 (1.2%)	− 0.05	0.47	9 (0.8%)	4 (0.8%)	0	0.99
Hypothyroidism	32 (2.8%)	25 (3.5%)	− 0.04	0.54	32 (2.8%)	8 (1.6%)	0.07	0.20
Disease severity								
APACHE II score	15 [11–18]	15 [11–19]	− 0.07	0.51	15 [11–18]	15 [11–18]	0.03	0.87
SOFA score	6 [4–8]	6 [4–8]	− 0.04	0.74	6 [4–8]	6 [4–8]	0.04	0.49
Pulmonary infiltrates			0.02	0.78			0.07	0.20
≤ 2	355 (31.8%)	223 (31.1%)			355 (31.8%)	131 (28.4%)		
> 2	762 (68.2%)	495 (68.9%)			762 (68.2%)	332 (71.6%)		
ARDS				0.005				0.22
Mild^d^	267 (23.9%)	209 (29.1%)	− 0.12		266 (23.8%)	110 (23.8%)	0	
Moderate^e^	566 (50.7%)	367 (51.1%)	− 0.01		566 (50.7%)	253 (54.6%)	− 0.08	
Severe^f^	284 (25.4%)	142 (19.8%)	0.13		285 (25.5%)	100 (21.6%)	0.09	
Laboratory data								
C-reactive protein (mg/dl)	15.9 [9.1–24.6]	17.1 [10.1–24.8]	− 0.09	0.09	16 [9.0–24.3]	17.4 [10.3–24.7]	− 0.06	0.11
Procalcitonin (ng/ml)	0.3 [0.2–0.8]	0.3 [0.2–0.9]	− 0.05	0.22	0.3 [0.2–0.8]	0.3 [0.2–0.7]	0.06	0.28
D-dimer (ng/ml)	1810 [790–4845]	1600 [742–4193]	0.09	0.03	1801 [790–4817]	1650 [774–4237]	0.13	0.24
Complications								
Shock	545 (48.8%)	370 (51.5%)	− 0.05	0.27	545 (48.8%)	242 (52.2%)	− 0.07	0.24
Acute kidney injury				0.01				0.81
RIFLE I	94 (8.4%)	84 (11.7%)	− 0.12		94 (8.4%)	38 (8.2%)	0.01	
RIFLE II	81 (7.3%)	67 (9.3%)	− 0.08		81 (7.3%)	31 (6.6%)	0.02	
RIFLE III	147 (13.2%)	101 (14.1%)	− 0.03		147 (13.2%)	54 (11.7%)	0.04	
Myocardial dysfunction	89 (7.8%)	91 (12.7%)	− 0.17	0.001	89 (7.8%)	37 (8.1%)	− 0.01	0.99
Bacterial co-infection	128 (11.4%)	74 (10.3%)		0.48	128 (11.4%)	49 (10.6%)		0.67
Treatments								
Remdesivir	24 (2.2%)	19 (2.7%)		0.59	24 (2.2%)	12 (2.6%)		0.72
Tocilizumab	392 (35.1%)	156 (21.7%)	0.28	< 0.001	392 (35.1%)	147 (31.7%)	0.07	0.21

Data are presented as numbers (%) or medians [interquartile range]. Smd < 0.01 are expressed as 0.01

*Smd* standardized mean differences, *BMI* body mass index, *ICU* intensive care unit, *ACE* angiotensin-converting enzyme, *ARBs* angiotensin receptor blockers, *COPD* chronic obstructive pulmonary disease, *APACHE* Acute Physiology And Chronic Health Evaluation, *SOFA* Sequential Organ Failure Assessment, *ARDS* acute respiratory distress syndrome, *RIFLE criteria* Risk, Injury, Failure, Loss, End stage

^a^Diagnosis gap means the time from disease onset to the confirmation of the diagnosis of SARS-CoV-2 infection

^b^Hospital gap means the time from disease onset to hospital admission

^c^ICU gap means the time from hospital to ICU admission

^d^Classified as the worst value of PaO_2_/FiO_2_ ratio < 300 within the first day of ICU admission

^e^Classified as the worst value of PaO_2_/FiO_2_ between 200 and 300 within the first day of ICU admission

^f^Classified as the worst value of PaO_2_/FiO_2_ < 100 within the first day of ICU admission

### Details in corticosteroid use

Among patients treated with corticosteroids, methylprednisolone was the most frequently administered (*n* = 856/1117, 76.6%), followed by dexamethasone (*n* = 247/1117, 22.1%). The time from onset of symptoms to corticosteroids initiation was 9 (IQR 7–12) days. The duration of treatment with methylprednisolone was 5 (IQR 3–10) days, whereas dexamethasone treatment was 10 (IQR 5–10) days (Table 2). In the multivariable analysis, factors independently associated with corticosteroid use were severe ARDS (OR 1.29; 95% CI 1.00–1.65; *p* = 0.04) and tocilizumab therapy (OR 1.88; 95% CI 1.50–2.35; *p* < 0.001; Additional file 1: Table S5). The rate of co-adjuvant therapies was similar between groups, except for lopinavir plus ritonavir and interferon which were more used in the No corticosteroid group (Additional file 1: Table S6).

**Table 2:** Description of corticosteroid use for COVID-19-associated acute respiratory distress syndrome patients

	Corticosteroid group (*n* = 1117)
Type of corticosteroid	
Methylprednisolone	856 (76.6%)
Dexamethasone	247 (22.1%)
Hydrocortisone	10 (0.9%)
Methylprednisolone plus hydrocortisone	2 (0.2%)
Methylprednisolone plus dexamethasone	2 (0.2%)
Timeline of corticosteroids use (days)	
Time from symptom onset to corticosteroid initiation	9 [7–12]
Duration of corticosteroid treatment	6 [3–10]
Methylprednisolone	5 [3–10]
Dexamethasone	10 [5–10]
ARDS severity when corticosteroid initiation	
Mild^a^ ARDS	267 (23.9%)
PaO_2_/FiO_2_ (mmHg)	240 [217–266]
Moderate^b^ ARDS	566 (50.7%)
PaO_2_/FiO_2_ (mmHg)	145 [121–169]
Severe^c^ ARDS	284 (25.4%)
PaO_2_/FiO_2_ (mmHg)	80 [69–91]

Data are expressed as numbers (%) or medians [interquartile range]

*ARDS* acute respiratory distress syndrome, *PaO*_*2*_*/FiO*_*2*_ partial pressure of oxygen to fractional inspired oxygen

^a^Classified as the worst value of PaO_2_/FiO_2_ ratio ≤ 300 to 200 mmHg within the first day of ICU admission

^b^Classified as the worst value of PaO_2_/FiO_2_ between < 200 and 100 mmHg within the first day of ICU admission

^c^Classified as the worst value of PaO_2_/FiO_2_ < 100 mmHg within the first day of ICU admission

### Mortality analysis

Overall, ICU mortality was 33.5% (*n* = 615/1835). In the multilevel logistic regression, no centre effect was observed for ICU mortality as between-centre variability was negligible (inter-hospital variance 0.17, SD ± 0.41, ICC 0.05). In the matched cohort, the ICU mortality did not differ between groups (corticosteroids *n* = 378/1117 [33.8%] vs. no corticosteroids *n* = 143/463 [30.9%]; *p* = 0.28). After adjusting for confounding factors (Additional file 1: Table S7), corticosteroid treatment was not independently associated with ICU mortality in the matched cohort (OR 1.26, 95% CI 0.96–1.65; *p* = 0.09; Additional file 1: Table S8).

**Fig. 2:**
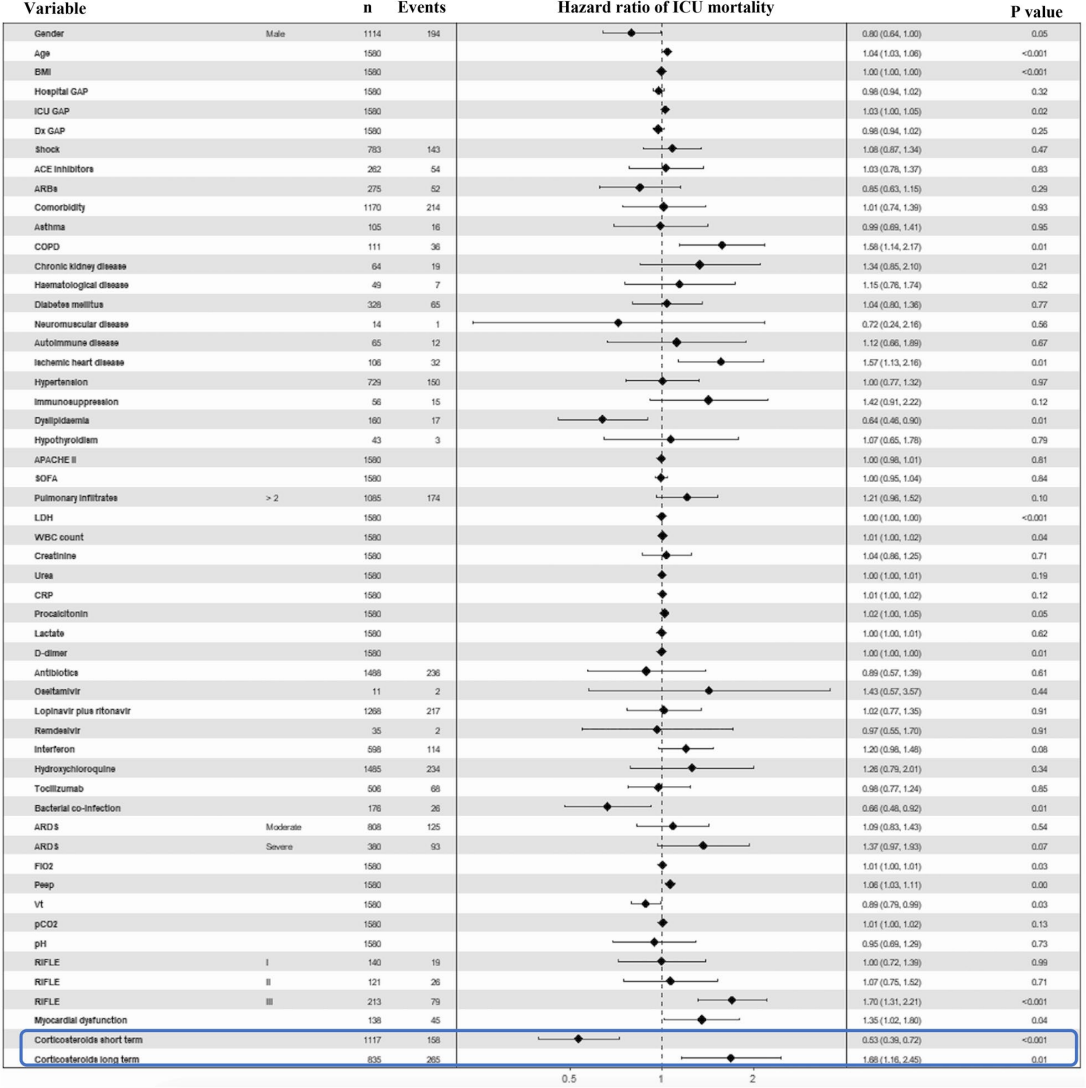
Forest plot of the cause-specific hazard model (time-dependent Cox regression) for ICU mortality. The time-dependent Cox regression included all the variables and those were adjusted as potential confounding factors. The interaction with time in this case was modelled using the step function to deal with non-proportional hazards of the covariate of interest (corticosteroid treatment). Through the step function, a partitioning of the time axis was made at day 17th, when the effect of the covariate changed over time. Consequently, the model allowed to estimate the effect of the treatment covariate before (corticosteroid treatment short-term) and after (corticosteroid treatment long-term) the 17th day of ICU admission. Notably, the regression model showed the protective effects of corticosteroids on survival up to day 17 of admission to the ICU, although these effects changed over time, as after the 17th day corticosteroid treatment at ICU admission was associated with higher mortality. Diagnosis GAP means the time (days) from disease onset to the confirmation of the diagnosis of SARS-CoV-2 infection; Hospital GAP (days) means the time from disease onset to hospital admission. ICU GAP (days) means the time from hospital to ICU admission. *BMI* body mass index, *ACE* angiotensin-converting enzyme, *ARBs* angiotensin receptor blockers, *COPD* chronic obstructive pulmonary disease, *APACHE* Acute Physiology And Chronic Health Evaluation, *SOFA* Sequential Organ Failure Assessment, *LDH* lactate dehydrogenase, *WBC* white blood cells count, *CRP* C-reactive protein, *ARDS* acute respiratory distress syndrome, *FiO*_*2*_ fraction of inspired oxygen, *Peep* positive end-expiratory pressure, *Vt* tidal volume, *pCO*_*2*_ partial pressure of carbon dioxide, *RIFLE criteria* Risk, Injury, Failure, Loss, End stage

**Fig. 3:**
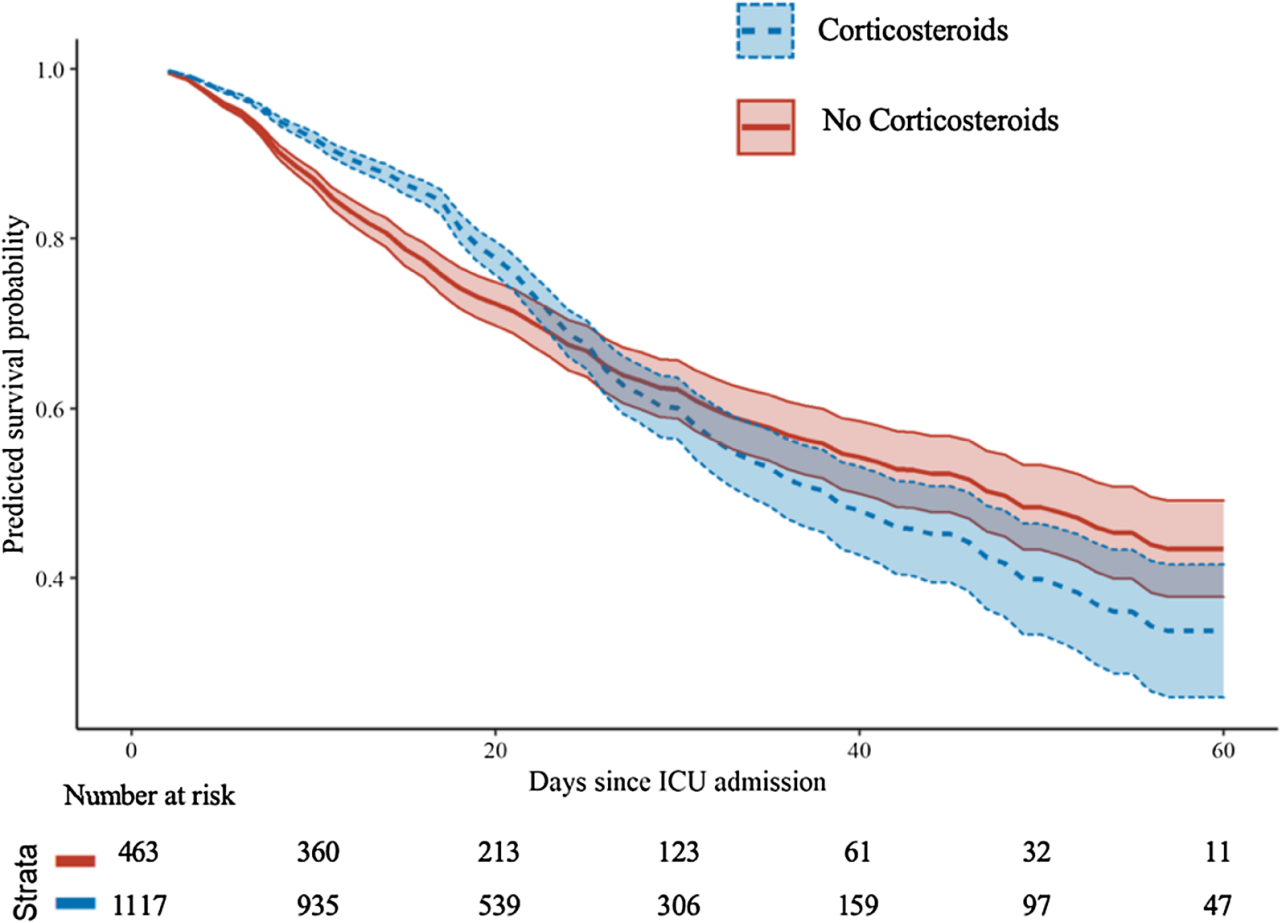
Survival analysis with the cause-specific hazard model and step function (time-dependent Cox regression) to investigate the association of corticosteroids and ICU mortality over time. The estimated survival curves showed the estimated effect of exposure of corticosteroids on ICU mortality over time. Survival curves cross each other implying time-dependency of corticosteroid exposure; hence step function approach was used to handle non-proportional hazards assumption by stratification of time on day 17 of follow-up when hazards changed the direction statistically significant. Then, the proportional hazards assumption was met for both periods. Models were adjusted for gender, age, body mass index, hospital GAP, ICU GAP, diagnosis GAP, shock, ACE inhibitors, ARBs, Comorbidity, asthma, COPD, chronic kidney disease, haematological disease, diabetes mellitus, neuromuscular disease, autoimmune disease, ischaemic heart disease, hypertension, immunosuppression, dyslipidaemia, hypothyroidism, APACHE II, SOFA, pulmonary infiltrates, lactate dehydrogenase, white blood cells count, creatinine, urea, C-reactive protein, procalcitonin, Lactate, d-dimer, antibiotics, oseltamivir, lopinavir plus ritonavir, remdesivir, interferon, hydroxychloroquine, Tocilizumab, bacterial co-infection, ARDS severity, fractional of inspired oxygen (FiO_2_), positive end-expiratory pressure, tidal volume, partial pressure of carbon dioxide, pH, RIFLE criteria, myocardial dysfunction and corticosteroid treatment (short and long-term). *ICU* intensive care unit, *ACE* angiotensin-converting enzyme, *ARBs* angiotensin receptor blockers, *COPD* chronic obstructive pulmonary disease, *APACHE* Acute physiology and chronic health evaluation, *SOFA* sequential organ failure assessment, *ARDS* acute respiratory distress syndrome

To evaluate the ICU mortality over time, a survival analysis was performed though the cause-specific hazard model using the step function. After adjusting for several confounding factors (Fig. 2), the time-dependent Cox regression showed that corticosteroids were associated with decreased ICU mortality (short-term HR 0.53; 95% CI 0.39–0.72; *p* < 0.001) within the 17th day of ICU admission but, beyond this timeframe the effect switched and corticosteroids were associated with increased ICU mortality thereafter (long-term HR 1.68; 95% CI 1.16–2.45; *p* = 0.01; Fig. 3). The Cox model for the competitive event (ICU discharged alive) is shown in the Additional file 1: Fig. S5.

**Fig. 4:**
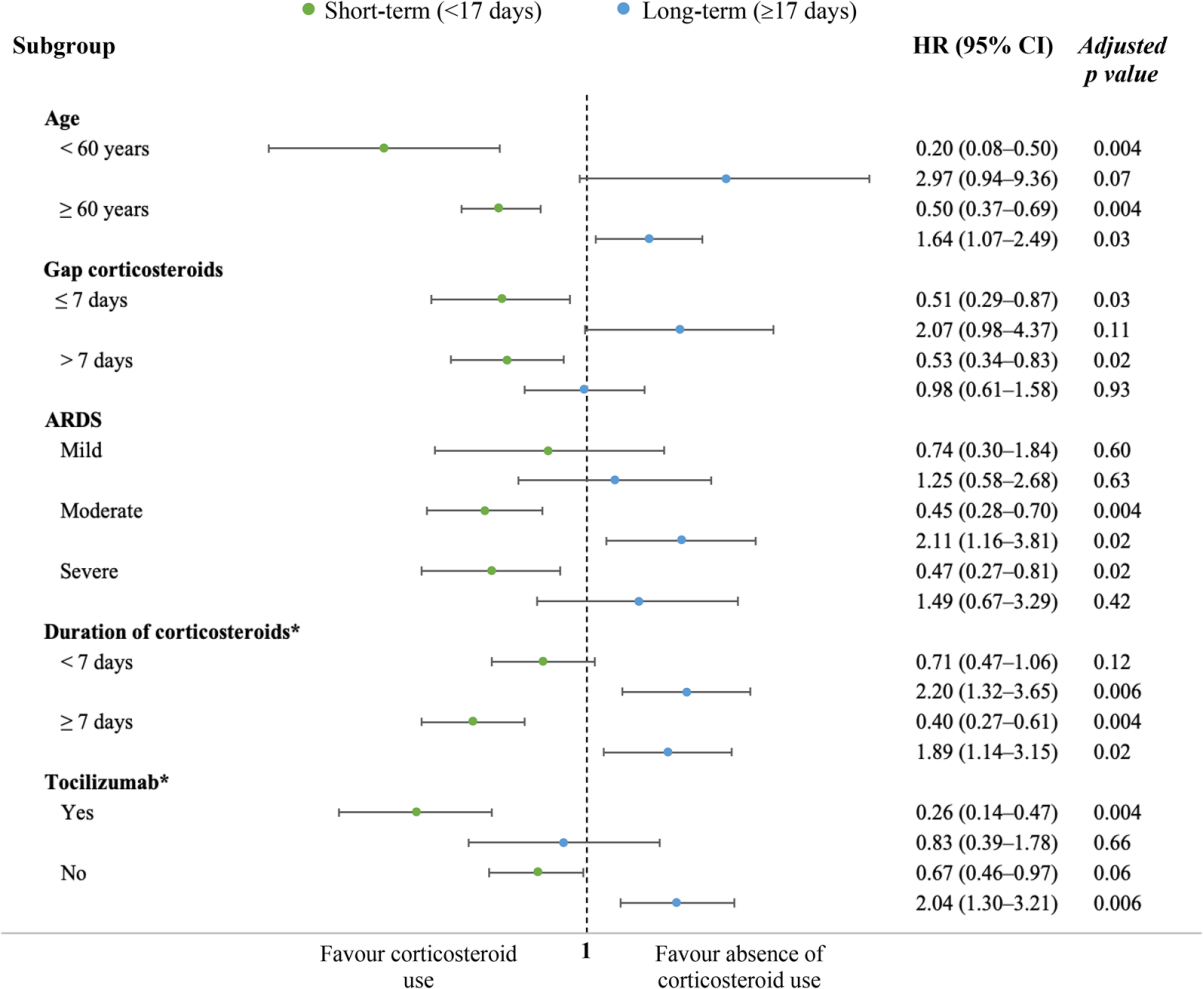
Subgroup sensitivity analysis. Propensity score matching was performed for each study subgroup as in the primary analysis to evaluate the estimated effect of corticosteroids on ICU mortality over time with cause-specific hazard model. Post hoc analysis was made for subgroups of duration of corticosteroid treatment and Tocilizumab. GAP corticosteroids mean the time (in days) since onset of symptoms to corticosteroid initiation. Models were adjusted for gender, age, body mass index, hospital GAP, ICU GAP, diagnosis GAP, shock, ACE inhibitors, ARBs, Comorbidity, asthma, COPD, chronic kidney disease, haematological disease, diabetes mellitus, neuromuscular disease, autoimmune disease, ischaemic heart disease, hypertension, immunosuppression, dyslipidaemia, hypothyroidism, APACHE II, SOFA, pulmonary infiltrates, lactate dehydrogenase, white blood cells count, creatinine, urea, C-reactive protein, procalcitonin, Lactate, d-dimer, antibiotics, oseltamivir, lopinavir plus ritonavir, remdesivir, interferon, hydroxychloroquine, Tocilizumab, bacterial co-infection, ARDS severity, fractional of inspired oxygen (FiO_2_), positive end-expiratory pressure, tidal volume, partial pressure of carbon dioxide, pH, RIFLE criteria, myocardial dysfunction and corticosteroid treatment (short and long-term). *ICU* intensive care unit, *ACE* angiotensin-converting enzyme, *ARBs* angiotensin receptor blockers, *COPD* chronic obstructive pulmonary disease, *APACHE* acute physiology and chronic health evaluation, *SOFA* sequential organ failure assessment, *ARDS* acute respiratory distress syndrome, *CRP* C-reactive protein

The sensitivity subgroup analysis with comparison of proportions of ICU mortality yielded similar finding as in the primary analysis, without finding significant differences between mortality rates across all the subgroups (Additional file 1: Table S9). In the survival analysis of subgroups, the time-dependent effect of corticosteroid treatment was also observed among the subgroups in the sensitivity analysis (Fig. 4 and Additional file 1: Table S10). Nonetheless, corticosteroids were associated with short-term survival benefit without long-term negative effects in subgroups of age < 60 years and severe ARDS, whereas no survival effects were found in mild ARDS (Additional file 1: Fig. S6). We further investigate the association of corticosteroids with mortality according with the duration of treatment (post hoc subgroup analysis in Additional file 1: Fig. S7). With shorter course of treatment (< 7 days), no survival effects were found in short-term, but associated with increased ICU mortality in long-term. When corticosteroid treatment lasted 7 days or longer, time-dependent effects were also found as in the main analysis. In a second post hoc analysis, patients who received corticosteroids plus tocilizumab presented short-term benefit on survival without long-term negative effects (Additional file 1: Fig. S8).

### Secondary outcomes

Secondary outcomes are shown in Table 3. In-hospital mortality was 38.3% in the corticosteroid group and 33.0% in the no corticosteroid group (*p* = 0.05). The number of ventilator-free days at 28 days did not achieve statistical differences and the cumulative incidence of ventilator-free days was also similar between groups after competing risks analysis (Additional file 1: Fig. S9). No differences were found in median ICU LOS in survivors. However, among non-survivors, the time to death was significantly larger in those who received corticosteroids compared with the non-corticosteroid users both in ICU (median 19 [IQR 11–29] vs. 12 [6–23], *p* < 0.001) and in hospital (median 24 [IQR 15–33] vs. 14 [IQR 9–24], *p* < 0.001). Regarding respiratory superinfection, corticosteroids were not associated with the development of VAP in the multivariable analysis (OR 1.05; 95% CI 0.83–1.34; Additional file 1: Table S11).

**Table 3:** Summary of the secondary outcomes of the study

	Corticosteroids (*n* = 1117)	No corticosteroids (*n* = 463)	*P* value
Secondary outcomes			
In-hospital mortality	428 (38.3%)	153 (33.0%)	0.05
Hospital LOS (days)			
Overall	30 (21–46)	30 (18–46)	0.63
Survivors	37 (25–53)	38 (25–52)	0.70
Non-survivors	24 (15–33)	14 (9–24)	< 0.001
Ventilator-free days at 28 days	8.2 ± 9.2	7.5 ± 8.6	0.17
ICU LOS (days)			
Overall	19 (11–31)	17 (10–31)	0.04
Survivors	18 (12–33)	20 (12–31)	0.82
Non-survivors	19 (11–29)	12 (6–23)	< 0.001
Ventilator-associated pneumonia	225 (20.1%)	90 (19.4%)	0.83

Ventilator-free days are expressed as mean and standard deviation using the Wilcoxon rank sum test with continuity correction. LOS (length of stay) is expressed as medians with interquartile ranges. Ventilator-associated pneumonia incidences are presented as numbers with percentages

## Discussion

In this study, corticosteroid treatment did not reduce ICU mortality rates among mechanically ventilated COVID-19-associated ARDS. However, our findings suggested that corticosteroids at ICU admission had time-dependent effects based on the short-term benefit of survival in the first 2 weeks, but an increased risk of mortality thereafter. Subgroups of patients aged less than 60 years, severe ARDS and tocilizumab plus corticosteroids showed short-term protective effects without deleterious long-term effects on mortality suggesting that not all mechanically ventilated COVID-19-associated ARDS patients might be corticosteroid-responsiveness. These results have been observed in a large cohort of ICU patients after controlling for observational-related biases and competing risks, thereby the benefit or potential harm of corticosteroids should be taken into account.

Data regarding the effectiveness of corticosteroids in COVID-19-associated ARDS remain limited. A recent meta-analysis (18 RCTs enrolling 2826 patients) evaluating the use of corticosteroids in ARDS (due to COVID-19 and non-COVID-19) concluded that corticosteroids probably reduce mortality in patients with ARDS of any aetiology [23]. However, the pooled estimate data of COVID-19-associated ARDS either using the ARDS criteria (risk ratio 0.92, 95% CI 0.76–1.11) and data from mechanically ventilated patients (risk ratio 0.89, 95% CI 0.71–1.12) did not reach statistical significance for 28-day mortality in the random-effects model. Different meta-analysis of COVID-19 patients with high heterogeneity on illness severity reported beneficial effects on mortality with corticosteroid treatment [7, 24, 25]. It should be pointed out that in most meta-analysis, the weight of the RECOVERY trial [3] made important contribution to overall pooled data. Conversely, conflicting results from other meta-analysis did not report benefits on mortality with corticosteroid treatment [8, 26].

The results of the RECOVERY trial [3] indicate that moderate dose of dexamethasone reduces 28-day mortality among patients with COVID-19 that require oxygen support, with more pronounced effect in those receiving invasive mechanical ventilation (rate ratio 0.64; 95% CI 0.51–0.81). This landmark trial has changed clinical practice worldwide for severe COVID-19 patients. Nonetheless, some uncertain issues deserve discussion and they must to be accounted for [6]. One major concern is that at day 28 of treatment allocation, 75% of patients were still hospitalized and investigating long-term outcomes could impact on the observed results [27]. Several factors affecting the outcome such as the level of respiratory support and the degree of hypoxemia were not measured, for instance, we found that corticosteroids may have higher benefit among severe ARDS, whereas patients with mild ARDS were not corticosteroid-responders. Additionally, there were 1707 (15%) patients who were not considered suitable for randomization but reasons for exclusion were not adequately disclosed. Physicians may exclude those patients because observed contraindications in whom corticosteroids might have detrimental consequences, which constitutes an important selection bias. Likewise, centre effect was not evaluated and there might be some imbalance in group allocation, especially in the subset of ventilated patients in which an average of only six patients were included per centre. Also in the stratified analysis, there was no benefit from dexamethasone in some subgroups, even in those with higher baseline risk (≥ 45%) which involves one half of the study patients. Therefore, the beneficial effect of corticosteroids among whole mechanically ventilated patients may be questionable.

The CoDEX trial performed in 299 ventilated COVID-19 moderate-to-severe ARDS assigned to received dexamethasone plus SOC versus SOC alone, did not find significant differences on all-cause 28-day mortality [28]. A randomized double-blind placebo-controlled trial conducted in 393 COVID-19 patients allocated to receive methylprednisolone 0.5 mg/kg twice daily for 5 days or placebo found no differences in 28-day mortality between groups, whereas lower mortality with corticosteroids was observed in post hoc analysis among patients over 60 years old (HR 0.63, 95% CI 0.41–0.97) [29]. Nonetheless, the rate of invasive mechanical ventilation was low and information regarding the degree of respiratory support or ARDS severity are lacking.

Results from retrospective also reported survival benefit for critically ill patients with COVID-19. In a multicentre cohort including 882 patients and high rates of invasive mechanical ventilation, it was observed that early corticosteroid treatment (within the first 48 h of ICU admission) was associated with a reduction in ICU mortality compared with delayed use or none after inverse probability weighting, although competing risks were not accounted for [30]. Retrospectives studies with robust statistical approach also showed positive effects with corticosteroids on mortality in severe [31] or COVID-19-associated ARDS [32], albeit smaller sample sizes and lower rates of invasive mechanical ventilation were recognized and these results might not be applicable to all COVID-19-associated ARDS. On the contrary, conflicting data from multicentre studies in COVID-19-associated ARDS [9, 10] and critical cases [11] suggested that corticosteroid treatment might be associated even with higher mortality, after carefully controlling for biases.

All this controversy on the evidence may be due to the heterogeneity of patients leading to conflicting data and it is unclear whether the use of corticosteroids is adequate for overall mechanically ventilated COVID-19 patients. We focused on mechanically ventilated patients with COVID-19-associated ARDS and observed that corticosteroid treatment at ICU admission had time-dependent effects. While corticosteroids seemed to be protective within the first 2 weeks of severe illness, the likelihood of survival changes beyond this timeframe and corticosteroid exposure at ICU admission appeared to be harmful. The possible explanations for the observed long-term negative effects from corticosteroids must be evaluated in further research as data regarding side effects are still lacking, since evidence from a recent meta-analysis suggested that there is no association between corticosteroids and superinfections [33], although a trend toward higher rates of delayed viral shedding and venous thromboembolism have been observed in some data [7, 26, 34–36].

Our results have been observed in a representative cohort of COVID-19-associated ARDS, with close similarities from other studies in terms of severity degree of hypoxemia, ventilatory parameters, and respiratory support management [37, 38]. The survival benefit from dexamethasone in mechanically ventilated patients in the RECOVERY trial [3] might be due to the capacity of corticosteroids to dampen both inflammation and late-onset fibrosis [39] in some cases that leads to severe lung injury in ARDS. Actually, in the present study, the subgroup of severe ARDS could benefit from corticosteroids over time without experimenting detrimental effects in long-term. Conversely, in patients with mild ARDS, corticosteroids had no significant effect on mortality questioning the need to treat such subgroup. These short-term protective effects have also been found for the subgroup of patients aged < 60 years, similar findings as those observed in the RECOVERY trial which showed protective effects of corticosteroids for patients younger than 70 years. However, a multicentre study conducted in 303 critically ill COVID-19 patients found opposite results to ours, reporting that early corticosteroid administration was associated with a lower mortality rate in patients aged ≥ 60 years [40]. The contradictory results might be due to a different population than ours with only one-third of included patients under invasive mechanical ventilation.

Regarding the duration of corticosteroid treatment, we found that a shorter course of treatment did not have protective effects, similar results as reported in the Metcovid trial [29]. Indeed, a meta-analysis found that ARDS patients (COVID-19 and non-COVID-19) who received a longer course of corticosteroids (over 7 days) had higher survival rates compared with a shorter course of treatment [23]. Despite the short-term benefit on survival with a longer course of treatment, possible long-term negative effects could arise; albeit, these results would need confirmation.

The use of tocilizumab plus corticosteroids at ICU admission seem to have also short-term protective effects on survival without presenting long-term deleterious effects on mortality. These findings coincide with a recent RCT conducted in hospitalized COVID-19 patients with hypoxia and systemic inflammation, in which the allocation to tocilizumab was associated with a significant reduction in 28-day mortality compared with usual care alone (with corticosteroid use up to 80% with systemic corticosteroids in both arms) [41].

Moreover, different phenotypes of COVID-19 critically ill patients with different host response patterns and impact on outcomes have been recently reported [42, 43]. Indeed, a retrospective study conducted in 428 critically ill COVID-19 patients reported that corticosteroids had significant survival benefits only in hyperinflammatory phenotype (HR 0.51; 95% CI 0.34–0.78) compared with the hypoinflammatory phenotype [44]. The subgroup analysis in our study showed that these time-dependent effects of corticosteroids on survival could be present in different subsets suggesting that patients’ response to corticosteroids may vary depending on distinct baseline conditions, although the exploratory nature of the sensitivity analysis requires that these results should be evaluated in further clinical research.

To our knowledge, this is one of the largest multicentre observational study of mechanically ventilated COVID-19-associated ARDS evaluating the effectiveness of corticosteroids on ICU mortality and results pointed out the possible time-dependent pattern of corticosteroids with likely opposite short and long-term effects on mortality. These findings support the hypothesis that not all mechanically ventilated patients with COVID-19-associated ARDS treated with corticosteroids have survival benefit over time. Our approach sought to account for selection bias and confounding, immortal time bias, and competing risks. In addition, performing subgroup sensitivity analysis made our results to be robust. As corticosteroids have become the SOC for severe COVID-19 patients, to randomize patients allocated to receive or not corticosteroids to investigate knowledge gaps would entail some ethical issues. This study attempted to make causal effects between corticosteroids and mortality from observational data. Hence, it provides real-world evidence on this topic currently under debate and new insights for a better personalization of care among mechanically ventilated patients with COVID-19-associated ARDS.

Nevertheless, our study had some limitations. First, as it was a retrospective study and some biases can arise from the observational design. Although RCTs are known to be the “gold standard” for research in investigating the effectiveness of interventions, methodological tools applied to observational data can decrease bias and confounding caused by the lack of randomization and could provide data from usual clinical practice. However, unmeasured confounders may persist. Second, our study was based on mechanically ventilated COVID-19-associated ARDS patients. Therefore, results may not be generalized to other settings such us hospitalized or outpatients. Third, we could not assess the established dosage of corticosteroids, although we deemed that suggested recommendations were followed using moderate doses of methylprednisolone, as all included patients in this study were diagnosed with ARDS [45]. Preferably, we focused on the effect of the exposure to corticosteroids at ICU admission. Nonetheless, dosage should be also a point of discussion in further research. Likewise, the safety profile and side effects such as hyperglycaemia, secondary superinfections different from respiratory source, or myopathy could not be evaluated. Fourth, different treatment approaches were used during the first wave, in accordance with current guidance [46], which could affect outcomes. Nevertheless, to avoid confounding by indication, all treatments received at admission were included within the adjusted Cox model after propensity score matching. Fifth, we did not collect data regarding the causes of death and the possibility of withdrawal of therapy beyond the 17th day of ICU admission. The population potentially at risk for life support withdrawal could be larger among corticosteroids users, therefore, some residual confounding may exist. Intensivists are used to facing to ethical concerns in their daily practice [47], and our ARDS patients died under invasive mechanical ventilation and other life support therapies in both groups, meaning that the most probable cause of death was persistent organ failure, mainly terminal respiratory failure [48]. Indeed, therapeutic withdrawal decisions in such critical care patients usually are taken under refractory/irreversibility stages of the disease. Further, we found that among non-survivors, the time to death was significantly larger in those who received corticosteroids compared with the non-corticosteroid users, suggesting that withdrawal life support rates might be lower in patients who received corticosteroids. Sixth, although two hundred and fifty-five patients without corticosteroids were not retained after matching, a sub-analysis revealed that no significant differences were found regarding demographic characteristics, severity of the disease, comorbidities and mortality, compared with those patients without corticosteroids included in the matching model. Nonetheless, propensity score matching yielded well balanced groups in terms of several observed confounders and, therefore eliminating selection bias. Seventh, conducting multiple testing for subgroup analysis may result in multiplicity. However, the sensitivity subgroup analysis was performed with an exploratory rather than confirmatory nature and these results aimed to yield consistency to the primary analysis and also to generate new hypothesis on corticosteroid-responsive subgroups for future research. Likewise, we accounted for multiplicity with the Benjamini–Hochberg approach to avoid the potential inflation of type I error. Eight, we could not evaluate the impact on outcomes of the ICU demand in periods of inundated critical care system. Finally, we did not collect data about viral shedding. The possibility of delay in viral clearance due to corticosteroid administration is an area for concern and more data are required to address this issue.

## Conclusion

In this study, corticosteroids treatment did not reduce ICU mortality rates among mechanically ventilated COVID-19-associated ARDS patients. However, our results suggested that corticosteroid treatment could present with biphasic time-dependent effects on survival with initial protective effects within the first 2 weeks of critical illness while beyond this timeframe there may be long-term negative effects. Subgroup of patients aged less than 60 years, severe ARDS and tocilizumab plus corticosteroids could be some of the subsets with the greatest benefit from corticosteroids, hence further research is needed to identify the treatment-responsive subgroups among mechanically ventilated COVID-19-associated ARDS patients.

## Supplementary Information

**Additional file 1.** Additional data of the study.

## Data Availability

The datasets used and/or analysed during the current study are available from the corresponding author on reasonable request.

## Abbreviations

APACHE: Acute Physiology and Chronic Health Evaluation
ARDS: Acute respiratory distress syndrome
CI: Confidence interval
COVID-19: Coronavirus disease 2019
HR: Hazard ratio
ICU: Intensive care unit
IQR: Interquartile range
LOS: Length of stay
OR: Odds ratio
PaO_2_/FiO_2_: Partial pressure of oxygen in arterial blood to fraction of inspired oxygen ratio
PEEP: Positive end-expiratory pressure
RCT: Randomized controlled trial
SARS-CoV-2: Severe acute respiratory syndrome coronavirus 2
SD: Standard deviation
SEMICYUC: Spanish Society of Intensive Care Medicine and Coronary Units
SOFA: Sequential Organ Failure Assessment
SOC: Standard of care
VAP: Ventilator-associated pneumonia
