# Supplementary material for: Corticosteroid treatment and mortality in mechanically ventilated COVID-19-associated acute respiratory distress syndrome (ARDS) patients: a multicentre cohort study
Source: Ann Intensive Care. 2021 Nov 26;11:159. doi: 10.1186/s13613-021-00951-0 (PMC8617372; doi:10.1186/s13613-021-00951-0)
Supplement: Supplementary file 1 — Additional file 1. Additional data of the study. [file 13613_2021_951_MOESM1_ESM.pdf]

Additional file 1

Corticosteroid treatment and mortality in mechanically ventilated COVID-19-associated acute respiratory distress syndrome (ARDS) patients: a multicentre cohort study

Gerard Moreno, MD (1); Raquel Carbonell, MD (1); Ignacio Martin-Loeches, PhD (2); Jordi Solé-Violán, PhD (3); Eudald Correig i Fraga, MS (4); Josep Gómez, PhD (1,5); Manuel Ruiz-Botella, MsC (1,5); Sandra Trefler, PhD (6); María Bodí, PhD (6); Josefa Murcia Paya, MD (7); Emili Díaz, PhD (8); Pablo Vidal-Cortes, MD (9); Elisabeth Papiol, MD (10); Antonio Albaya Moreno, PhD (11); Susana Sancho Chinesta, MD (12); Lorenzo Socias Crespi, PhD (13); María del Carmen Lorente, PhD (14); Ana Loza Vázquez, MD (15); Rebeca Vara Arlanzon, MD (16); María Teresa Recio, MD (17); Juan Carlos Ballesteros, MD (17); Ricard Ferrer, PhD (18); Elisabeth Fernandez Rey, MD (19); Marcos I Restrepo, PhD (20); Ángel Estella, PhD (21); Antonio Margarit Ribas, MD (22); Neus Guasch, MD (22); Luis F Reyes, PhD (23); Judith Marín-Corral, PhD (24); and Alejandro Rodríguez, PhD (6) on behalf of the COVID-19 SEMICYUC Working Group*

Table of contents

1. **Additional methods**
2. **Additional tables**
3. **Additional figures**
4. **COVID-19 SEMICYUC Working group investigators**

1. Additional methods

Data Collection and Validation

Data were collected using a paper CRF (case Report Form). CRF collect and record all protocol-required information, which is transcribed from patient source documents, such as hospital records and laboratory data during the patient's participation in the study. Before sending the CRF to the Study Coordinator (AR), these data were de-identified by removing the patient's name, medical record number, etc., and giving the patient a unique study number. We implemented a double data entry model for potential errors in real-time. Data was entered twice by two different Data Entry personnel based on the same set of data collected in the paper CRFs. All data were reviewed, and values that appeared incongruent or out of range were manually validated by confirming the accuracy of the data with the Study Coordinator (AR). The database was validated and cleaned before the statistical analysis and finally, the study database was locked to prevent any further changes, and to ensure data consistency and integrity for the statistical reporting and analysis.

Study definitions

The confirmation of case of SARS-CoV-2 infection was accomplished by positive reverse transcription-polymerase chain reaction, either at hospital or ICU admission, from specimens collected with nasopharyngeal and oropharyngeal swabs according to the WHO recommendations [1]. Lower respiratory tract specimens were collected when patients were under MV and high clinical suspicion was present, if previous tests were negatives.

Shock at ICU admission was defined as the requirement of any dose of vasopressor therapy within the first six hours of admission to maintain appropriate main blood pressure, despite adequate fluid resuscitation targeted by dynamic hemodynamic parameters and/or echocardiography.

Acute kidney injury was defined according the RIFLE criteria [2].

Community-acquired respiratory co-infection (CARC) or bacterial co-infection was considered in patients with confirmation of SARS-CoV-2 infection showing recurrence of fever, increase in cough and production of purulent sputum plus positive bacterial/fungal respiratory or blood cultures within the first two days of ICU admission [3].

Statistical analysis

Missing data were handled with multiple imputation by chained equations [4]. All patients considered in the study received Corticosteroids within the first day of ICU stay at the latest. Time zero of follow up was ICU admission, but we discarded all patients censored within 48h of ICU admission to avoid immortal time bias.

Genetic matching (GM) was used to reduce treatment selection bias and balance the covariance matrix for both groups. GM uses a genetic search algorithm to iteratively determine the weight of each of the covariates to find an optimal balance between matched groups [5]. The matching was one to one with replacement and ties (so that one treated unit could be matched to more than one untreated unit after weighting them appropriately) and without calipers. Variables included in the propensity score matching model were those baseline variables related to the outcome, as recommended elsewhere [6].

The matching weights were included as case weights in the Cox procedures (*see Therneau, T., Grambsch, P., Modeling Survival Data: Extending the Cox Model. Springer-Verlag, 2000., page 161 for more information.*).

Centre effect for ICU mortality was investigated by multilevel logistic regression analysis through a conditional random intercept model using inter-hospital variation as a random-effects variable. Regression coefficients were summarized as the variance with standard deviation (SD) and the interclass correlation coefficient (ICC). The ICC can be interpreted as "the proportion of the variance explained by the grouping structure in the population". This index goes from 0, if the grouping conveys no information, to 1, if all observations in a group are identical. When ICC is large, it means the between-class variance cannot be ignored and therefore a multilevel model is preferred. It has been suggested that if ICC > 0.1, one should consider the use of a multilevel model. When the ICC is not different from zero or negligible, one could consider running traditional one level regression analysis [7,8].

As a complementary analysis to the main outcome (ICU mortality), we conducted a survival analysis through a Cox regression to investigate whether survival times were related to covariates, and estimating the effect size of a corticosteroid treatment after adjusting for potential confounders. Survival analysis examines and models the time it takes for events to occur, then focuses on the distribution of survival times.

The Cox proportional-hazards model is essentially a regression model for investigating the association between the survival time of patients and one or more predictor variables and it permits to analyse how specified factors influence the rate (hazard rate) of a particular event happening (e.g., death) at a particular point in time. Therefore, the validity of the Cox regression model relies on the assumption of proportionality of the hazard rates of individuals which must remain constant over time with different covariates values [9]. Discharge alive from ICU has been identified as a competitive event for ICU mortality [10], then the survival analysis was performed using the Cox regression through a cause-specific hazard model [11,12]. This model enables the interpretation of etiological relationships and the estimation of effect between the exposure and both outcomes. An important assumption of survival analysis such as the Cox model is that censoring is 'independent'. This independent censoring assumption implies that patients who are censored at a certain time point should be representative for those still at risk (and thus in the risk set) at that point in time. However, this assumption cannot be made if, for example, the survival time of an individual is censored, being withdrawn as a result of a deterioration or an amelioration in his/her physical condition. This is probably the case in the ICU where patients are discharged alive, and thus withdrawn from the survival analysis, because they need no more intensive care, usually due to amelioration or deterioration of their vital conditions. Patients are therefore discharged alive (censored) because they have a lower risk or higher risk of hospital death than the average. These patients are therefore not the same patient population as those who stayed within the hospital. Resulting censoring is 'informative', meaning that censoring carries information about or depends on the survival time. In other words, informative censoring defined a competing risk, given that discharge from the hospital affects the probability of experiencing the event of interest (death before discharge). In this setting, standard survival methods are no longer valid, and specific methods need to be considered.

When the Cox model was made, the proportional hazards assumption was strongly violated for corticosteroids. Hence, the variable of interest (corticosteroids) was considered as a time-varying covariate, which occurs when a given covariate changes over time during the follow-up period, a common phenomenon in clinical research [13]. Proportional hazards may not hold over the entire time axis but may hold approximately over shorter time periods. The effect of a time-varying covariate (corticosteroid treatment) becomes stronger or weaker over time, which can be explored via stratification by time. Therefore, we carried out a time-dependent Cox regression using a step function to deal with non-proportional hazards [14]. The step function consisted in a partitioning of the time axis dividing the follow up into shorter time periods, hence the proportional hazard assumption held within each interval of the partition. We established to divide the study time frame in two intervals (at 17th of follow up) when the proportional hazards assumption was met. The rationale behind 17 days-step term was based on the pronounced change of case-fatality rates according with both the life-tables and the survival curves in this timeframe. With this methodology, we modelled the effect of corticosteroids on mortality in two ranges: the short and long-term. The coefficients for these two time periods have been estimated separately so that each covariate can relate differently to survival during the two time periods. The benefit of partitioning the time axis is that we can model the effect of the corticosteroid treatment for each period of the study. With the time split at 17 days of follow up, permitted to observed that the hazard rates of individuals of each interval were constant over time, hence, the assumption of proportionality was met. In this way we model the effect of Corticosteroids on mortality in two ranges: the short and the long-term. The results of time-to-event data were expressed as hazard ratios (HR) and 95% CI."

Prespecified subgroup sensitivity analysis with exploratory nature was performed with propensity score matching for each study subgroup to evaluate whether the observed effect of corticosteroids on ICU mortality was consistent across subgroups, and to assess the robustness of our findings. ICU mortality was investigated either by comparing proportions in the matched subsets and survival analysis with cause-specific hazard model. Subgroups were based on previous research as well as clinical relevance and categorized as: age (< 60, ≥ 60), severity of ARDS (mild, moderate and severe), and time since the symptom onset to the initiation of corticosteroids (< 7 days, ≥ 7days). To account for multiplicity and avoid the potential inflation of the type I error rate as a result of multiple testing in the subgroup analysis, we used the Benjamini-Hochberg method for controlling the false discovery rate [15,16].

Ventilator-free days were defined as follows: 28 – "x" if successfully weaned from mechanical ventilation "x" days after ICU admission; for subjects who died within 28 days of mechanical ventilation or who were mechanically ventilated for more than 28 days, ventilator-free days were coded as zero. Differences between groups were assessed by Wilcoxon rank sum test and reported as means and standard deviations. We also evaluated ventilator-free days with time-to-event analysis censored at 28 days, with the event of interest the successful liberation from the mechanical ventilation and mortality as a competitive event. We

used sub-distribution hazard analysis (Fine-Gray model) to address the situation when more than one end-points are possible. This analysis provides a sub-distribution hazard ratio which estimate the association of the effect and the outcome (successfully liberation from mechanical ventilation), accounting for the existence of an alternative outcome (ICU mortality) [17].

References

1. World Health Organization. Clinical management of severe acute respiratory infection (SARI) when COVID-19 disease is suspected. Interim guidance [Internet]. 2020. p. 9–26. Available from: https://apps.who.int/iris/handle/10665/331446
2. Bellomo R, Ronco C, Kellum JA, Mehta RL, Palevsky P. Acute renal failure - definition, outcome measures, animal models, fluid therapy and information technology needs: the Second International Consensus Conference of the Acute Dialysis Quality Initiative (ADQI) Group. Crit Care. 2004;8.
3. Martín-Loeches I, Sanchez-Corral A, Diaz E, Granada RM, Zaragoza R, Villavicencio C, et al. Community-acquired respiratory coinfection in critically III patients with pandemic 2009 influenza A(H1N1) virus. Chest. 2011;139:555–62.
4. White IR, Royston P, Wood AM. Multiple imputation using chained equations: Issues and guidance for practice. Stat Med. 2011;30:377–99.
5. Diamond A, Sekhon JS. Genetic matching for estimating causal effects: A general multivariate matching method for achieving balance in observational studies. Rev Econ Stat. 2013;95:932–45.
6. Brookhart MA, Schneeweiss S, Rothman KJ, Glynn RJ, Avorn J, Stürmer T. Variable selection for propensity score models. Am J Epidemiol. 2006;163:1149–56.
7. Sommet N, Morselli D. Keep calm and learn multilevel logistic modeling: A simplified three-step procedure using stata, R, Mplus, and SPSS. Int Rev Soc Psychol. 2017;30:203–18.
8. Kahan BC. Accounting for centre-effects in multicentre trials with a binary outcome - When, why, and how? BMC Med Res Methodol. BMC Medical Research Methodology; 2014;14:20.
9. Moriguchi S, Hayashi Y, Korenaga D. Patients With Cast r ic Cancer. J Surg Oncol. 1993;13:9–13.
10. Resche-Rigon M, Azoulay E, Chevret S. Evaluating mortality in intensive care units: Contribution of competing risks analyses. Crit Care. 2006;10:R5.
11. Austin PC, Lee DS, Fine JP. Introduction to the Analysis of Survival Data in the Presence of Competing Risks. Circulation. 2016;133:601–9.
12. Noordzij M, Leffondré K, Van Stralen KJ, Zoccali C, Dekker FW, Jager KJ. When do we need competing risks methods for survival analysis in nephrology? Nephrol Dial Transplant. 2013;28:2670–7.
13. Zhang Z, Reinikainen J, Adeleke KA, Pieterse ME, Groothuis-Oudshoorn CGM. Time-varying covariates and coefficients in Cox regression models. Ann Transl Med. 2018;6:121–121.
14. Ahmed FE, Vos PW, Holbert D. Modeling survival in colon cancer: A methodological review. Mol Cancer. 2007;6:1–12.
15. Benjamini Y, Hochberg Y. Controlling The False Discovery Rate - A Practical And Powerful Approach To Multiple. 1995;57:289–300.
16. Menyhart O, Weltz B, Gyorffy B. Multipletesting.com: A tool for life science researchers for multiple hypothesis testing correction. PLoS One. 2021;16:e0245824.
17. Yehya N, Harhay MO, Curley MAQ, Schoenfeld DA, Reeder RW. Reappraisal of ventilator-free days in critical care research. Am J Respir Crit Care Med. 2019;200:828–36.

2. Additional tables

Table S1. SROBE Statement checklist

	Item No.	Recommendation	Section or Page in the manuscript
**Title and abstract**	1	(*a*) Indicate the study's design with a commonly used term in the title or the abstract	Title
		(*b*) Provide in the abstract an informative and balanced summary of what was done and what was found	Abstract
**Introduction**			
Background/rationale	2	Explain the scientific background and rationale for the investigation being reported	Introduction
Objectives	3	State specific objectives, including any prespecified hypotheses	Introduction, last paragraph
**Methods**			
Study design	4	Present key elements of study design early in the paper	Methods
Setting	5	Describe the setting, locations, and relevant dates, including periods of recruitment, exposure, follow-up, and data collection	Methods
Participants	6	(*a*) *Cohort study*—Give the eligibility criteria, and the sources and methods of selection of participants. Describe methods of follow-up	Methods
		(*b*) *Cohort study*—For matched studies, give matching criteria and number of exposed and unexposed	Methods, statistical analysis
Variables	7	Clearly define all outcomes, exposures, predictors, potential confounders, and effect modifiers. Give diagnostic criteria, if applicable	Methods
Data sources/ measurement	8*	For each variable of interest, give sources of data and details of methods of assessment (measurement). Describe comparability of assessment methods if there is more than one group	Methods
Bias	9	Describe any efforts to address potential sources of bias	Methods (statistical analysis) and limitation section in the discussion
Study size	10	Explain how the study size was arrived at	No sample size was calculation
Quantitative variables	11	Explain how quantitative variables were handled in the analyses. If applicable, describe which groupings were chosen and why	Methods
Statistical methods	12	(*a*) Describe all statistical methods, including those used to control for confounding	Methods
		(*b*) Describe any methods used to examine subgroups and interactions	Methods
		(*c*) Explain how missing data were addressed	Methods
		(*d*) *Cohort study*—If applicable, explain how loss to follow-up was addressed	Methods
		(*e*) Describe any sensitivity analyses	Methods and supplementary appendix
**Results**			
Participants	13*	(a) Report numbers of individuals at each stage of study—e.g. numbers potentially eligible, examined for eligibility, confirmed eligible, included in the study, completing follow-up, and analyzed	Results, first paragraph
		(b) Give reasons for non-participation at each stage	Results first paragraph
		(c) Consider use of a flow diagram	Figure 1
Descriptive data	14*	(a) Give characteristics of study participants (e.g. demographic, clinical, social) and information on exposures and potential confounders	Results, second paragraph
		(b) Indicate number of participants with missing data for each variable of interest	Supplement
		(c) *Cohort study*—Summaries follow-up time (e.g., average and total amount)	Methods

Outcome data	15*	*Cohort study*—Report numbers of outcome events or summary measures over time	Results
Main results	16	(*a*) Give unadjusted estimates and, if applicable, confounder-adjusted estimates and their precision (e.g., 95% confidence interval). Make clear which confounders were adjusted for and why they were included	Results
		(*b*) Report category boundaries when continuous variables were categorized	Results
		(*c*) If relevant, consider translating estimates of relative risk into absolute risk for a meaningful time period	Not applicable
Other analyses	17	Report other analyses done—e.g. analyses of subgroups and interactions, and sensitivity analyses	Results, subgroup section
**Discussion**			
Key results	18	Summaries key results with reference to study objectives	Discussion
Limitations	19	Discuss limitations of the study, taking into account sources of potential bias or imprecision. Discuss both direction and magnitude of any potential bias	Limitation section at the end of the discussion
Interpretation	20	Give a cautious overall interpretation of results considering objectives, limitations, multiplicity of analyses, results from similar studies, and other relevant evidence	Discussion and Limitation section
Generalizability	21	Discuss the generalizability (external validity) of the study results	Limitation section
**Other information**			
Funding	22	Give the source of funding and the role of the funders for the present study and, if applicable, for the original study on which the present article is based	At the end of the methods

Table S2. Full list of data collected during the study.

**General characteristics**
Hospital type (According to beds number: <200, 200–500 and >500)
Gender (male/female)
Age
Body mass index
Date of symptom onset
Date of Hospital admission
Date of ICU admission
Date of ICU discharge
Data of Hospital discharge
GAP ICU (time between hospital to ICU admission, days)
GAP diagnosis (time to symptom onset to diagnosis confirmation, days)
GAP antiviral treatment (time from onset of symptoms to first dose of antiviral, days)
**Illness severity**
APACHE (Acute Physiology and Chronic Health Evaluation) II score
Sequential Organ Failure Assessment (SOFA) score
ARDS (acute respiratory distress syndrome): Mild, moderate or severe.
Number of pulmonary infiltrates on chest X-ray
**Comorbidities**
Asthma
Chronic Pulmonary Obstructive Disease
Arterial Hypertension
Dyslipidaemia
Obesity (BMI > 30 Kg/m^2)
Diabetes mellitus
Ischemic heart disease
Chronic Heart disease (New York Heart Association (NYHA) Functional Classification III and IV)
Chronic kidney disease (Estimated Glomerular Filtration Rate (eGFR) < 60 $ml/min/1.73m^2$)
Haematological disease (acute leukaemia, myelodysplastic syndrome and lymphomas)
Pregnancy
HIV/AIDS (Human immunodeficiency virus infection and acquired immunodeficiency syndrome)
Neuromuscular disease
Immunological disease
Hypothyroidism
Other underlying conditions
**Laboratory findings**
D-Lactate dehydrogenase (LDH)
White blood cell (WBC) count
Creatinine
C-Reactive Protein (CRP)
Procalcitonin (PCT)
Lactate
D-dimer
Ferritin

Arterial blood gas test
**Treatment at ICU admission**
Corticosteroids (type of corticosteroid, indication and duration)
Antibiotics
Lopinavir/ritonavir
Hydroxychloroquine
Tocilizumab
Interferon β
Remdesivir
Prior anti-hypertensive treatment (ACE: Angiotensin Converting Enzyme Inhibitors. ARB: Angiotensin receptor blockers)
**Oxygenation and respiratory support within the first 24 hours of ICU admission**
Oxygen mask
High Flow nasal cannula
Non-invasive ventilation
Invasive mechanical ventilation
Ventilatory parameters:<br>Fraction of inspired oxygen (FiO_2)<br>Partial pressure of oxygen (PaO_2)<br>PaO_2/FiO_2 ratio<br>Positive end-expiratory pressure (PEEP)<br>Tidal volume (Vt)<br>Plateau pressure
**Complications at ICU admission**
Shock
Acute kidney injury (According to RIFLE criteria)
Myocardial dysfunction
Community-acquired co-infection (CARC)
**Outcomes**
ICU mortality
Length of ICU stay
Duration of mechanical ventilation
Ventilator-associated pneumonia (VAP)

Table S3. Data of missing values.

The remaining variables had no missing values. Ferritin, interleukin-6 and lymphocytes count with high missing data were not considered for primary analysis and were used only for descriptive analysis. ICU = intensive care unit; APACHE = Acute Physiology and Chronic Health Evaluation; SOFA = Sequential Organ Failure Assessment.

	**% missing data**
Body mass index	8
Time from symptom onset to hospital admission	11.3
Time from hospital admission to ICU admission	0.3
Time from symptom onset to diagnosis of COVID-19	11
Prior angiotensin converting enzyme inhibitors	0.2
Prior angiotensin receptor blockers	0.2
APACHE II score	5.4
SOFA score	12.5
Pulmonary infiltrates on chest X-ray	0.2
Lactate dehydrogenase	15.6
White blood cells count	1.5
Creatinine	1.6
Urea	1.5
C-reactive protein	5.6
Procalcitonin	17.2
D-dimer	17.7
Ferritin	72.4
Interleukin-6	83.9
Lymphocyte's count	77.8
Time from symptom onset to corticosteroid initiation	7.2
Type of corticosteroid	4.9
Duration of corticosteroid treatment	10.3
Partial pressure of oxygen to fraction of inspired oxygen (PaO_2/FiO_2) ratio	13.3
Plateau pressure	37.7
Positive end-expiratory pressure	8.4
Respiratory rate	17.5
Partial pressure of carbon dioxide	18.9
Tidal volume	28.7
pH	19.2
Duration of mechanical ventilation	7.5
Myocardial dysfunction	0.2
Acute renal injury	0.2
Prone position	0.3
Continuous renal replacement therapy	1.1
Extracorporeal membrane oxygenation	0.2

Table S4. Respiratory characteristics of study groups (corticosteroids vs no corticosteroids) stratified by the severity of ARDS in COVID-19 ventilated patients within the first day of ICU admission.

Data are numbers (%) or medians (interquartile range). ARDS = Acute respiratory distress syndrome; PaO_2/FiO_2 = arterial oxygen partial pressure to fractional inspired oxygen ratio; PEEP = positive end-expiratory pressure; RR = respiratory rate; pCO2 = partial pressure of carbon dioxide; V_t = tidal volume; PBW = Predicted body weight; ECMO = extracorporeal membrane oxygenation; MV = mechanical ventilation.

	**ARDS severity N=1835**								
	**Mild (n=476)**			**Moderate (n=933)**			**Severe (n=426)**		
**Variable**	**Corticosteroids (n=267)**	**No Corticosteroids (n=209)**	***P* value**	**Corticosteroids (n=566)**	**No Corticosteroids (n=367)**	***P* value**	**Corticosteroids (n=284)**	**No Corticosteroids (n=142)**	***P* value**
PaO_2/FiO_2 (mmHg)	240 (217–266)	240 (220–263)	0.67	145 (121–169)	150 (122–172)	0.15	80 (69–91)	81 (66–90)	0.85
FiO_2 (%)	60 (50–80)	50 (45–70)	<0.01	70 (60–100)	65 (50–80)	<0.01	100 (100–100)	100 (80–100)	0.06
PEEP (cmH_2O)	12 (10–14)	12 (10–14)	0.10	12 (10–14)	12 (10–14)	0.23	12 (10–14)	12 (10–14)	0.73
RR (bpm)	20 (17–22)	20 (18–22)	0.82	20 (18–23)	20 (18–23)	0.73	20 (17–23)	18 (17–22)	0.21
pH	7.36 (7.31–7.41)	7.36 (7.31–7.42)	0.55	7.36 (7.30–7.41)	7.37 (7.31–7.42)	0.15	7.35 (7.28–7.42)	7.33 (7.28–7.39)	0.37
pCO2 (mmHg)	43 (38–49)	41 (36–48)	0.05	44 (39–51)	43 (38–49)	0.08	45 (40–54)	46 (40–54)	0.91
V_T (mL/Kg PBW)	6.5 (6.0–7.0)	6.5 (6.0–7.4)	0.31	6.4 (6.0–7.0)	6.4 (6.0–7.0)	0.62	6.5 (6.0–7.3)	6.7 (6.0–7.2)	0.51
Plateau pressure (cmH_2O)	25 (22–28)	25 (22–28)	0.26	26 (23–29)	25 (22–28)	0.12	27 (23–30)	26 (23–30)	0.85
Prone position	172 (64.6%)	123 (58.8%)	0.21	347 (61.3%)	215 (58.5%)	0.40	213 (74.7%)	116 (81.6%)	0.12
ECMO	8 (3%)	4 (1.9%)	0.45	9 (1.6%)	7 (1.9%)	0.71	3 (1.1%)	1 (0.7%)	0.72
Recruitment manoeuvres	163 (61%)	96 (45.9%)	<0.01	306 (54%)	158 (43%)	<0.01	202 (71.1%)	85 (59.9%)	0.02
Duration of MV (days)	15 (8–25)	14 (8–25)	0.39	15 (9–27)	15 (9–26)	0.74	17 (9–28)	14 (8–25)	0.15

Table S5. Multivariable analysis for factors associated to corticosteroid treatment at ICU admission.

ARDS = acute respiratory distress syndrome; ICU = intensive care unit.

	OR	95% CI	*P* value
**Variable**			
Myocardial dysfunction	0.64	0.46–0.89	0.009
Acute renal failure	0.79	0.64–0.98	0.03
C-reactive protein > 15 mg/dl	0.80	0.65–0.98	0.03
Mild ARDS	0.82	0.65–1.04	0.10
D-dimer	1.00	1.00–1.00	0.12
Time from hospital admission to ICU admission	1.09	0.98–1.03	0.49
Severe ARDS	1.29	1.00–1.65	0.04
Tocilizumab	1.88	1.50–2.35	<0.001

Table S6. Treatments administrated at ICU admission in the matched cohort.

**Treatments**	**Corticosteroid group (n = 1117)**	**No Corticosteroids group (n = 463)**	***P* value**
Antibiotics	1062 (95.1%)	430 (92.9%)	0.11
Lopinavir plus ritonavir	872 (78.1%)	399 (86.2%)	<0.001
Hydroxychloroquine	1061 (95%)	436 (94.1%)	0.55
Interferon beta	364 (32.6%)	257 (55.6%)	<0.001
Tocilizumab	392 (35.1%)	147 (31.7%)	0.21
Remdesivir	24 (2.2%)	12 (2.6%)	0.72

Table S7. Comparison between survivors and non-survivors in the matched cohort.
Data are expressed as medians (IQR) or numbers (%). APACHE = Acute Physiology and Chronic Health Evaluation; SOFA = Sequential Organ Failure Assessment; ARDS = acute respiratory distress syndrome; ICU = Intensive Care Unit; COPD = Chronic Obstructive Pulmonary Disease; RIFLE criteria for the acute kidney injury = I Risk, II Injury, III Failure. VAP = Ventilator-associated pneumonia.

	**Matched cohort n = 1580**		
	**Survivors n = 1053**	**Non-survivors n = 527**	***P* value**
**Variable**			
**Demographics**			
Age (years)	62 (54-69)	69 (63-74)	<0.001
Gender (male)	723 (68.7%)	391 (74.2%)	0.02
**Illness severity**			
APACHE II score	14 (10-17)	17 (13-21)	<0.001
SOFA score	5 (4-7)	6 (5-8)	<0.001
ARDS			<0.001
Mild	293 (27.8%)	99 (18.8%)	
Moderate	542 (51.5%)	266 (50.5%)	
Severe	218 (20.7%)	162 (30.7%)	
**Disease timeline** (days)			
Time from symptom onset to hospital admission	7 (5-8)	6 (5-8)	<0.001
Time from hospital admission to ICU admission	2 (0-4)	2 (0-4)	0.88
**Comorbidities**			
Hypertension	431 (40.9%)	298 (56.5%)	<0.001
Diabetes mellitus	199 (18.9%)	129 (24.5%)	0.01
Ischemic heart disease	43 (4.1%)	63 (12%)	<0.001
Asthma	72 (6.8%)	33 (6.3%)	0.66
COPD	49 (4.7%)	62 (11.8%)	<0.001
Chronic kidney disease	31 (2.9%)	33 (6.3%)	0.002
Haematological disease	27 (2.6%)	22 (4.2%)	0.08
Immunosuppression	26 (2.5%)	30 (5.7%)	0.001
**Laboratory data**			
C-reactive protein (mg/dl)	16 (9-24)	18 (10-25)	0.03
D-dimer (ng/ml)	1490 (700-4002)	2354 (1026-6122)	<0.001
**Complications**			
Shock	487 (46.2%)	296 (56.2%)	<0.001
RIFLE criteria			<0.001
I	91 (8.6%)	49 (9.3%)	
II	63 (6%)	58 (11%)	
III	63 (6%)	150 (28.5%)	
Myocardial dysfunction	56 (5.3%)	82 (15.6%)	<0.001
Bacterial co-infection	115 (10.9%)	61 (11.6%)	0.69
VAP	194 (18.4%)	120 (22.8%)	0.04
**Treatments on ICU admission**			
Corticosteroids	739 (70.2%)	378 (71.7%)	0.52
Lopinavir plus ritonavir	827 (78.5%)	441 (83.7%)	0.02
Interferon beta-1a	374 (35.5%)	224 (42.5%)	0.007
Hydroxychloroquine	998 (93.8%)	497 (94.3%)	0.70
Tocilizumab	362 (34.4%)	144 (27.3%)	0.005
Remdesivir	27 (2.6%)	8 (1.5%)	0.18

Table S8. Multivariable logistic regression for associated factors with ICU mortality in the matched cohort.
ICU = Intensive care Unit; OR = odds ratio; CI = Confidence interval; APACHE = Acute Physiology and Chronic Health Evaluation; SOFA = Sequential Organ Failure Assessment; COPD = Chronic Obstructive Pulmonary Disease; ARDS = acute respiratory distress syndrome; RIFLE criteria for acute kidney injury = I Risk, II injury, III failure; VAP = ventilator-associated pneumonia.

	**OR**	**95% CI**	***P* value**
**Variable**			
Male gender	1.06	0.81 – 1.39	0.66
Age	1.06	1.04 – 1.07	<0.001
Hospital gap	0.95	0.91 – 0.98	0.002
APACHE II score	1.01	0.99 – 1.04	0.16
SOFA score	1.03	0.97 – 1.09	0.27
C-reactive protein	1.01	0.99 – 1.02	0.07
D-dimer	1.00	1.00 – 1.00	0.12
Shock at ICU admission	1.26	0.97 – 1.64	0.08
COPD	2.06	1.31 – 3.23	0.002
Chronic kidney disease	1.13	0.59 – 2.14	0.70
Haematological disease	2.02	1.04 – 3.95	0.04
Diabetes mellitus	1.12	0.83 – 1.52	0.43
Ischemic heart disease	1.94	1.21 – 3.11	0.006
Hypertension	1.08	0.83 – 1.40	0.52
Immunosuppression	1.89	1.02 – 3.51	0.04
Corticosteroids	1.26	0.96 – 1.65	0.09
Lopinavir plus ritonavir	1.20	0.85 – 1.68	0.28
Interferon beta	1.19	0.92 – 1.56	0.18
Tocilizumab	0.96	0.74 – 1.26	0.78
Mild ARDS	0.71	0.52 – 0.97	0.03
Severe ARDS	1.57	1.18 – 2.10	0.002
Myocardial dysfunction	1.70	1.11 – 2.58	0.01
RIFLE I	1.24	0.82 – 1.87	0.30
RIFLE II	2.03	1.32 – 3.13	0.001
RIFLE III	5.17	3.56 – 7.50	<0.001
VAP	1.03	0.76 – 1.38	0.84

Table S9. Subgroup analysis with comparison of proportion of the ICU mortality between Corticosteroid and No Corticosteroid in matched samples.

To account for multiplicity, "p" values were adjusted by the Benjamini-Hochberg method for controlling the false discovery rate. *Post-hoc analysis.

	ICU mortality			
**Matched subgroups**	**Corticosteroids** (nº deaths / total nº)	**No corticosteroids** (nº deaths / total nº)	***P***	**Adjusted *P value***
Age				
< 60 years	62/361 (17.2%)	16/149 (10.1%)	0.08	0.33
≥ 60 years	316/756 (41.8%)	133/314 (42.3%)	0.92	0.99
Gap corticosteroids				
≤ 7 days	129/332 (38.9%)	59/162 (36.1%)	0.62	0.85
> 7 days	191/653 (29.3%)	83/256 (32.3%)	0.40	0.62
ARDS				
Mild	69/266 (25.9%)	21/120 (17.6%)	0.09	0.33
Moderate	187/566 (33%)	66/239 (27.7%)	0.16	0.35
Severe	122/285 (42.8%)	40/97 (41%)	0.85	0.99
Corticosteroid treatment duration*				
< 7 days	245/683 (35.9%)	111/364 (30.5%)	0.09	0.33
≥ 7 days	133/434 (30.6%)	88/290 (30.3%)	0.99	0.99
Tocilizumab*				
Yes	108/392 (27.6%)	36/114 (31.6%)	0.40	0.62
No	270/725 (37.2%)	113/349 (32.4%)	0.12	0.33

Table S10. Subgroup analysis with time-dependent Cox regression and step function. Summary of hazard ratios accounting for time (short and long-term).
Analysis was performed with Cause-specific hazard model for both events accounting for the time-dependency effect of corticosteroid treatment with step function by partitioning time period of follow up on day 17th of follow up, as in the primary analysis. To account for multiplicity, "p" values were adjusted by the Benjamini-Hochberg method for controlling the false discovery rate. *Post-hoc analysis.
GAP corticosteroids mean the time since symptom onset to corticosteroid exposure.
Models were adjusted for gender, age, body mass index, hospital GAP, ICU GAP, diagnosis GAP, shock, ACE inhibitors, ARBs, Comorbidity, asthma, COPD, chronic kidney disease, haematological disease, diabetes mellitus, neuromuscular disease, autoimmune disease, ischemic heart disease, hypertension, immunosuppression, dyslipidaemia, hypothyroidism, APACHE II, SOFA, pulmonary infiltrates, lactate dehydrogenase, white blood cells count, creatinine, urea, C-reactive protein, procalcitonin, Lactate, D-dimer, antibiotics, oseltamivir, lopinavir plus ritonavir, remdesivir, interferon, hydroxychloroquine, Tocilizumab, bacterial co-infection, ARDS severity, fractional of inspired oxygen (FiO2), positive end-expiratory pressure, tidal volume, partial pressure of carbon dioxide, pH, RIFLE criteria, myocardial dysfunction and corticosteroid treatment (short and long-term). GAP corticosteroids mean the time (in days) since onset of symptoms to corticosteroid initiation. ICU = intensive care unit; ACE = angiotensin converting enzyme; ARBs = angiotensin receptor blockers; COPD = chronic obstructive pulmonary disease; APACHE = Acute physiology and chronic health evaluation; SOFA = sequential organ failure assessment; ARDS = acute respiratory distress syndrome; CRP = C-reactive protein.

	**Cause-specific hazard model ICU mortality**				**Cause-specific hazard model Discharge alive (competitive event)**			
	**Short-term <17 days**		**Long-term > 17 days**		**Short-term <17 days**		**Long-term >17 days**	
**Subgroups**	**HR (95% CI)**	***Adjusted P***	**HR (95% CI)**	***Adjusted P***	**HR (95% CI)**	***Adjusted P***	**HR (95% CI)**	***Adjusted P***
Age								
< 60 years	0.20 (0.08–0.50)	0.004	2.97 (0.94–9.36)	0.07	1.42 (0.98–2.06)	0.11	0.62 (0.44–0.87)	0.02
≥ 60 years	0.50 (0.37–0.69)	0.004	1.64 (1.07–2.49)	0.03	1.09 (0.77–1.54)	0.75	0.53 (0.40–0.70)	0.003
Gap corticosteroids								
≤ 7 days	0.51 (0.29–0.87)	0.03	2.07 (0.98–4.37)	0.11	0.65 (0.41–1.03)	0.11	0.81 (0.54–1.21)	0.38
> 7 days	0.53 (0.34–0.83)	0.02	0.98 (0.61–1.58)	0.93	1.33 (0.94–1.88)	0.15	0.60 (0.44–0.82)	0.003
ARDS								
Mild	0.74 (0.30–1.84)	0.60	1.25 (0.58–2.68)	0.63	0.74 (0.48–1.13)	0.22	0.42 (0.28–0.64)	0.003
Moderate	0.45 (0.28–0.70)	0.004	2.11 (1.16–3.81)	0.02	1.42 (0.96–2.09)	0.13	0.61 (0.44–0.85)	0.003
Severe	0.47 (0.27–0.81)	0.02	1.49 (0.67–3.29)	0.42	1.14 (0.63–2.09)	0.75	0.52 (0.31–0.85)	0.02
Corticosteroid duration*								
< 7 days	0.71 (0.47–1.06)	0.12	2.20 (1.32–3.65)	0.006	0.94 (0.67–1.31)	0.75	0.47 (0.34–0.64)	0.003
≥ 7 days	0.40 (0.27–0.61)	0.004	1.89 (1.14–3.15)	0.02	1.36 (1.01–1.84)	0.08	0.70 (0.53–0.92)	0.02
Tocilizumab*								
Yes	0.26 (0.14–0.47)	0.004	0.83 (0.39–1.78)	0.66	1.06 (0.64–1.76)	0.82	0.59 (0.38–0.90)	0.04
No	0.67 (0.46–0.97)	0.06	2.04 (1.30–3.21)	0.006	1.05 (0.80–1.39)	0.75	0.52 (0.40–0.68)	0.003

Table S11. Logistic regression analysis for factors associated with development of ventilator-associated pneumonia.

	OR	95% Confidence interval	*P* value
Corticosteroids	1.05	0.83–1.34	0.65
Gender (male)	1.24	0.95–1.67	1.11
Acute kidney injury	1.32	1.03–1.70	0.025
Prone position	2.17	1.62–2.90	<0.001
Haematological disease	1.72	0.99–2.97	0.05
Age	1.01	1.00–1.03	0.001

3. Additional figures

Figure S1. Hospital distribution of mechanically ventilated COVID-19-associated ARDS patients admitted in each participating Intensive Care Unit in the study.

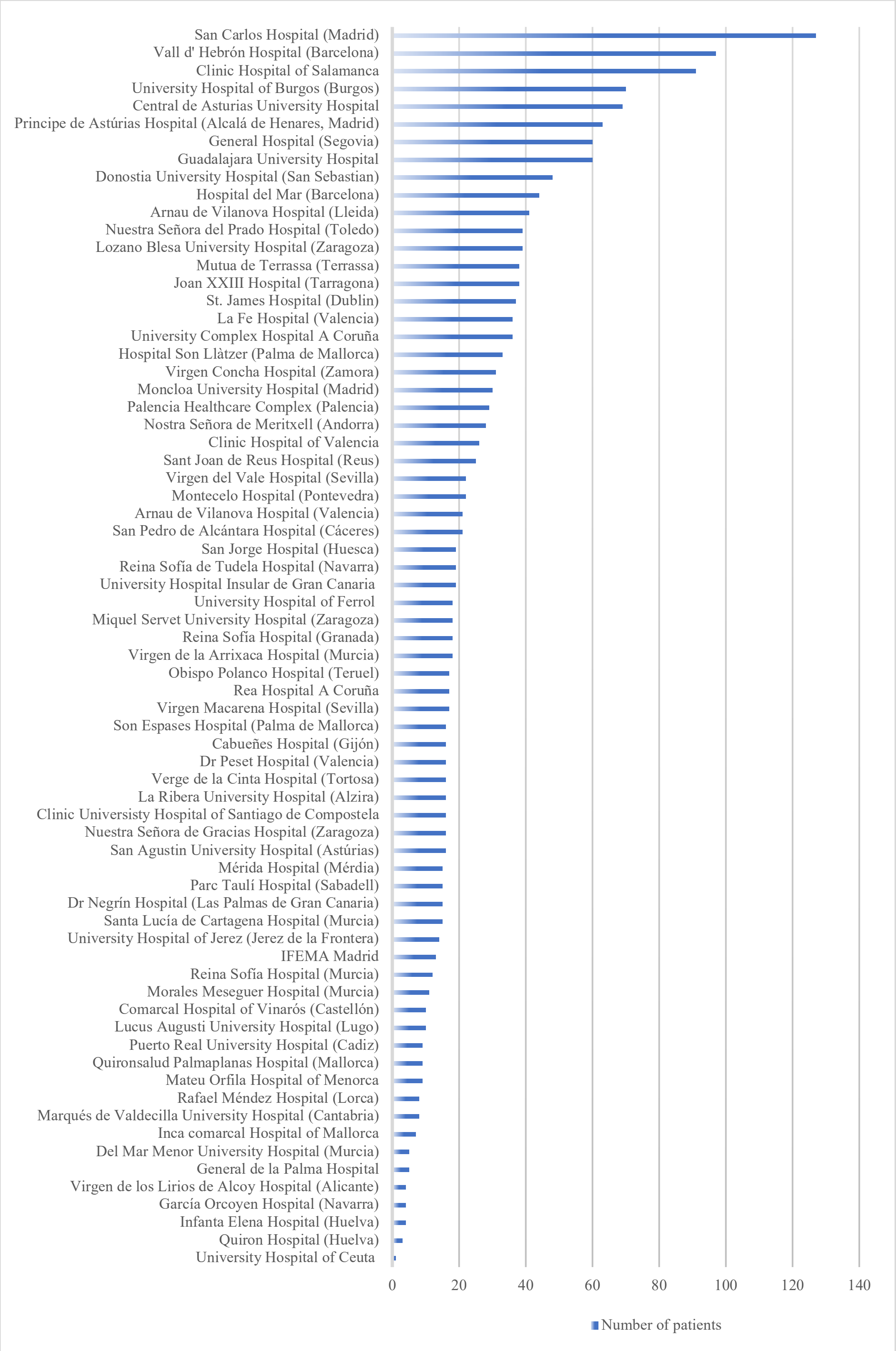

Figure S2. Time distribution of included cases in the study according with corticosteroid treatment (methylprednisolone, dexamethasone o none).
The fourteen remaining patients with corticosteroids received hydrocortisone (n=10) or combination of corticosteroids (n=4). Large number of patients were included during the first wave in Spain (March-April 2020).

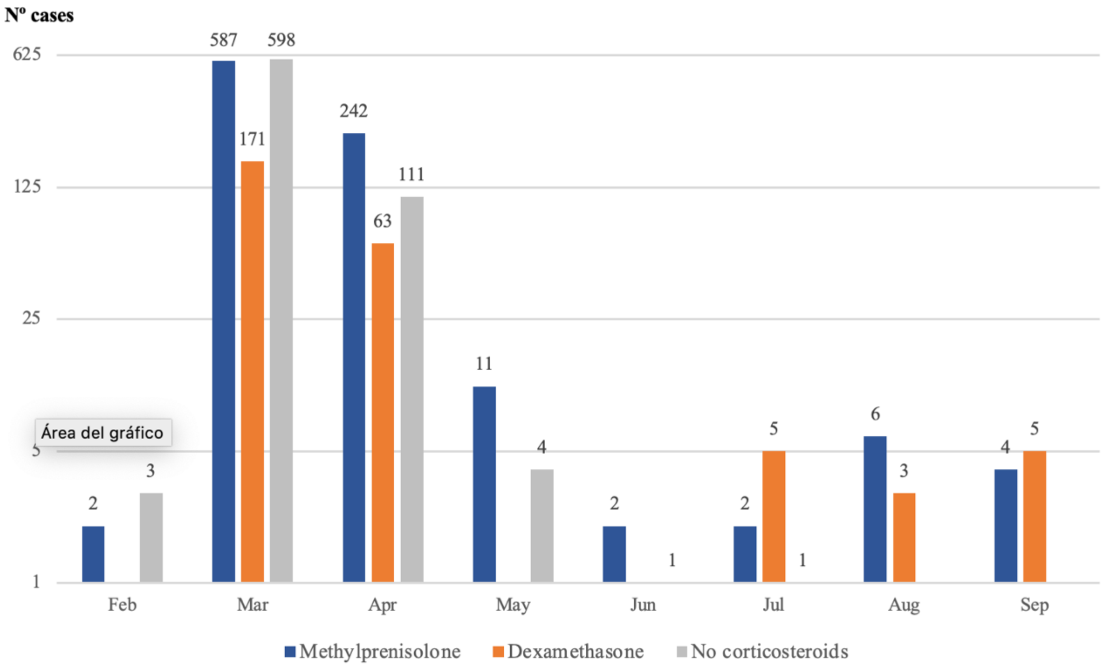

Figure S3. Schoenfeld Residual Plot for corticosteroid treatment.
The solid line (βt) gives the estimated effect of the predictor through time (with confidence intervals depicted as dashed lines). Significant violation of the assumption of proportional hazards were found for the corticosteroid treatment.

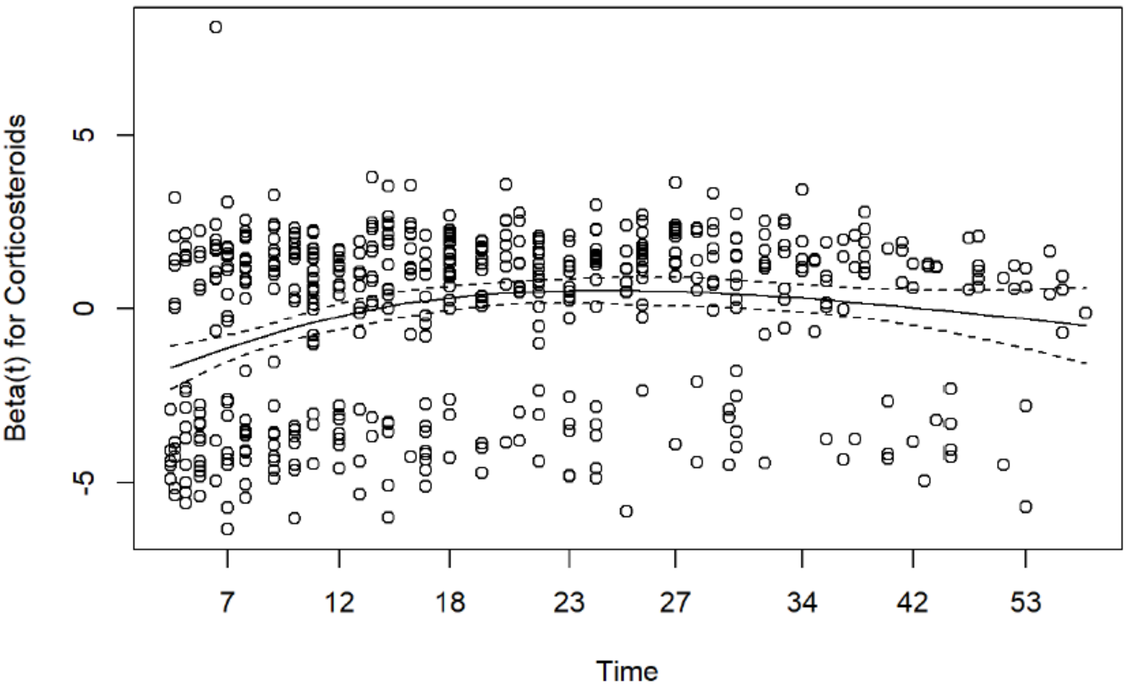

Figure S4. Love plot of the covariate balance of propensity score matching with unadjusted and adjusted standardized mean differences.

Hospital gap is the time period from symptoms onset to hospital admission. ICU gap is the time period from hospital to ICU admission. ICU = Intensive care unit; COPD = Chronic obstructive pulmonary disease; APACHE = Acute Physiology And Chronic Health Evaluation; SOFA = Sequential Organ Failure Assessment; ARDS = Acute Respiratory Distress Syndrome; RIFLE = RIFLE criteria for acute kidney injury (Risk, Injury, Failure, Loss, End stage).

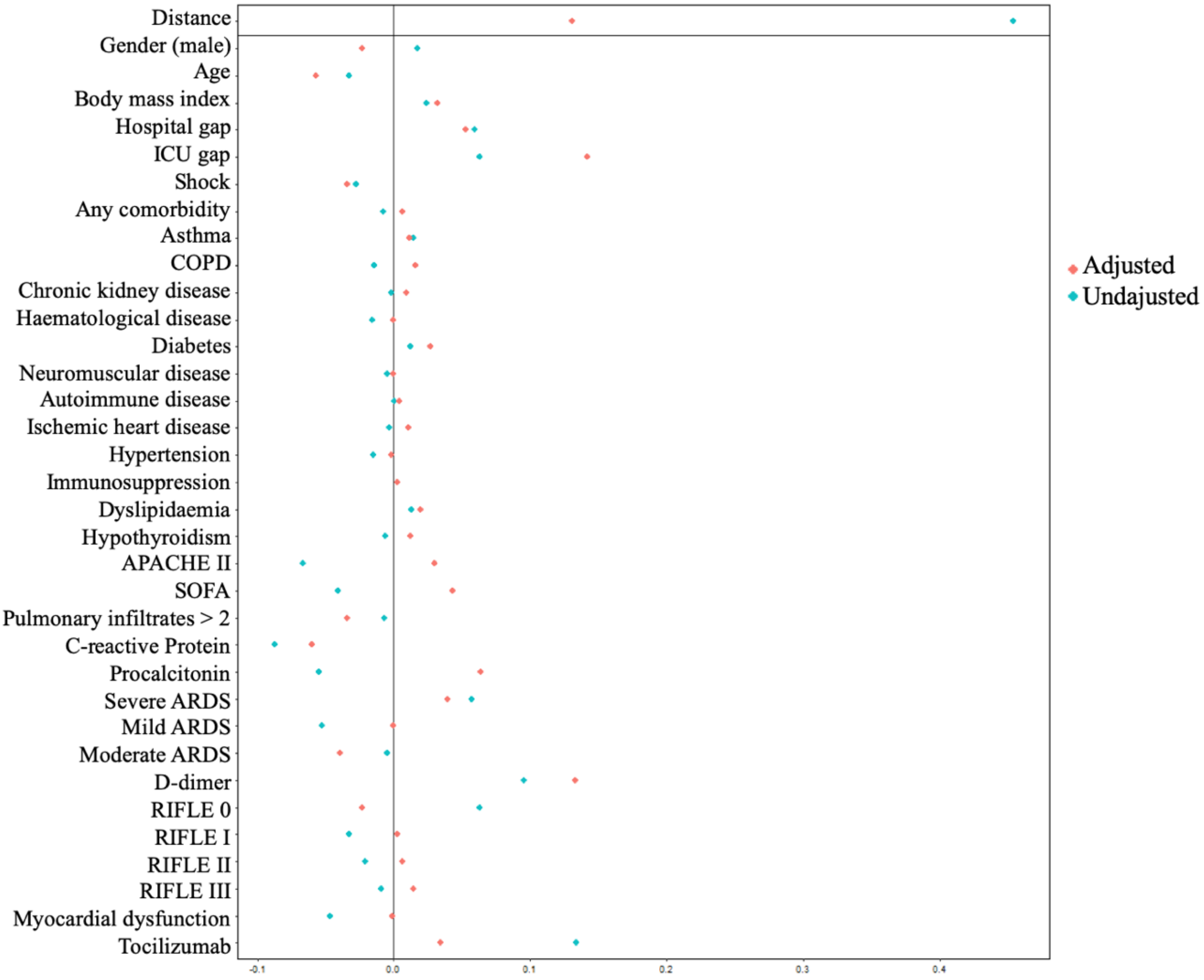

Figure S5. Forest plot of the Cause-specific hazard model for ICU discharged alive (competitive event of ICU mortality).

Corticosteroids were associated with lower probability of discharge alive in short-term, but exposure on admission in long-term was associated with higher probability of discharge alive.

BMI: body mass index. Diagnosis gap means the time (days) from disease onset to the confirmation of the diagnosis of SARS-CoV-2 infection. Hospital gap (days) means the time from disease onset to hospital admission. ICU gap (days) means the time from hospital to ICU admission. ACE = Angiotensin-converting enzyme; ARBs = Angiotensin receptor blockers; COPD = Chronic obstructive pulmonary disease; APACHE = Acute Physiology And Chronic Health Evaluation; SOFA = Sequential Organ Failure Assessment; LDH = lactate dehydrogenase; WBC = white blood cells count; CRP = C-reactive protein; ARDS = Acute respiratory distress syndrome; FiO_2: fraction of inspired oxygen; PEEP = positive end-expiratory pressure; Vt = tidal volume; pCO_2 = partial pressure of carbon dioxide; RIFLE criteria: Risk, Injury, Failure, Loss, End stage.

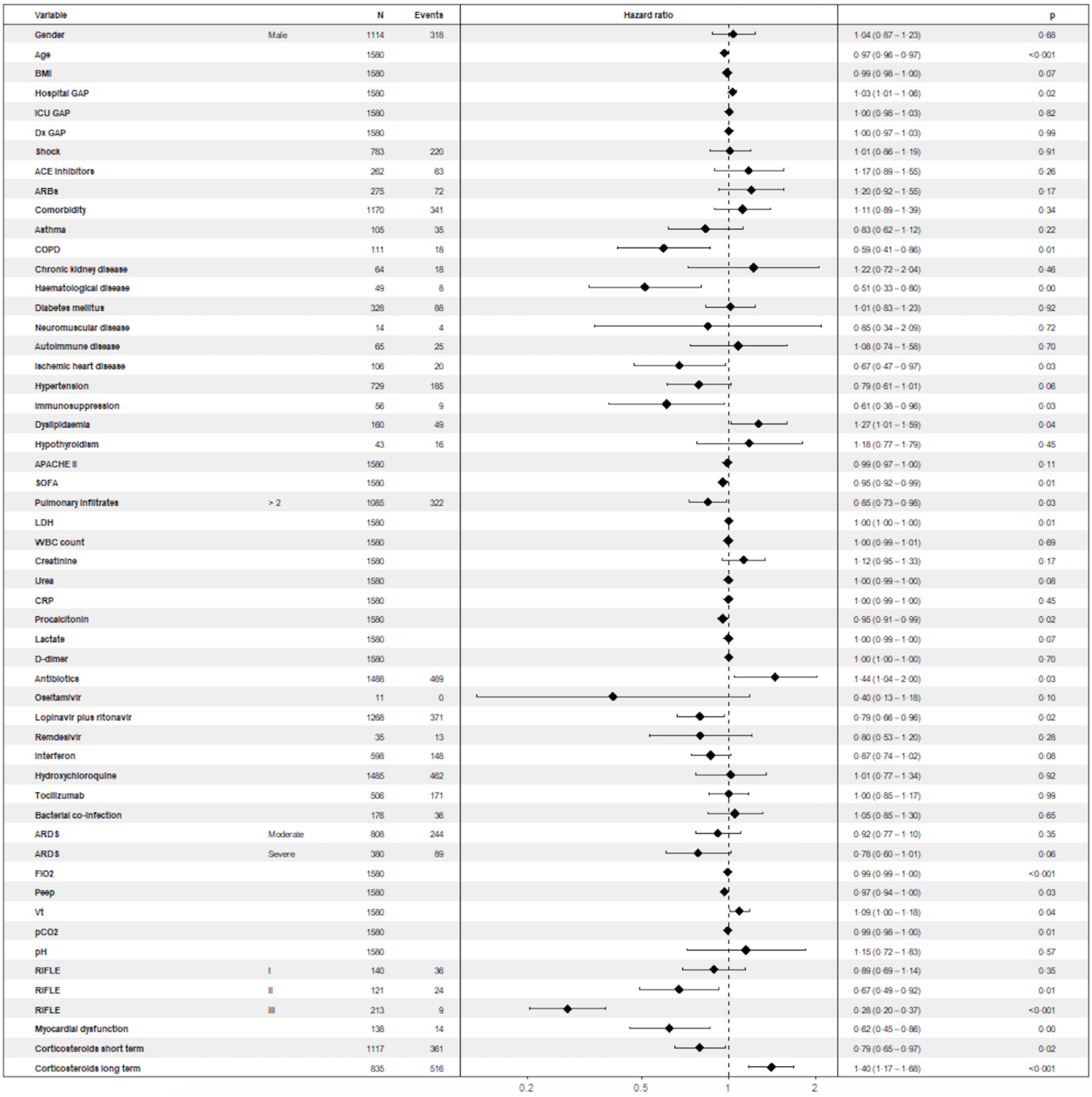

Figure S6. Pre-specified subgroups analysis. Survival plots of the Cause-specific hazard model for ICU mortality trough time-dependent Cox regression with step-function.

Adjusted for gender, age, body mass index, hospital GAP, ICU GAP, diagnosis GAP, shock, ACE inhibitors, ARBs, Comorbidity, asthma, COPD, chronic kidney disease, haematological disease, diabetes mellitus, neuromuscular disease, autoimmune disease, ischemic heart disease, hypertension, immunosuppression, dyslipidaemia, hypothyroidism, APACHE II, SOFA, pulmonary infiltrates, lactate dehydrogenase, white blood cells count, creatinine, urea, C-reactive protein, procalcitonin, Lactate, D-dimer, antibiotics, oseltamivir, lopinavir plus ritonavir, remdesivir, interferon, hydroxychloroquine, Tocilizumab, bacterial co-infection, ARDS severity, fractional of inspired oxygen (FiO2), positive end-expiratory pressure, tidal volume, partial pressure of carbon dioxide, pH, RIFLE criteria, myocardial dysfunction and corticosteroid treatment (short and long-term). ICU: intensive care unit, ACE: angiotensin converting enzyme, ARBs: angiotensin receptor blockers, COPD: chronic obstructive pulmonary disease, APACHE: Acute physiology and chronic health evaluation, SOFA: sequential organ failure assessment, ARDS: acute respiratory distress syndrome.

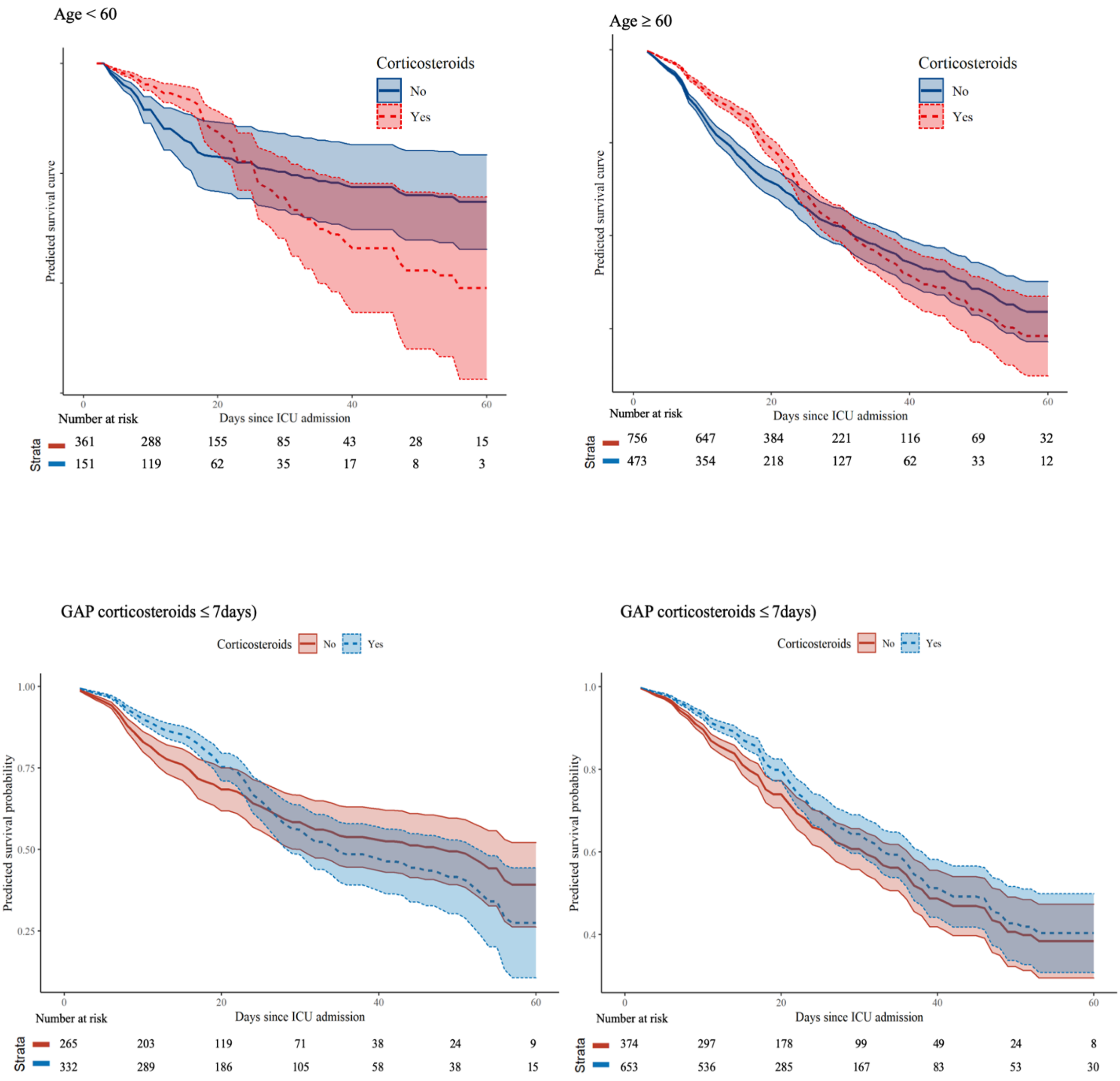

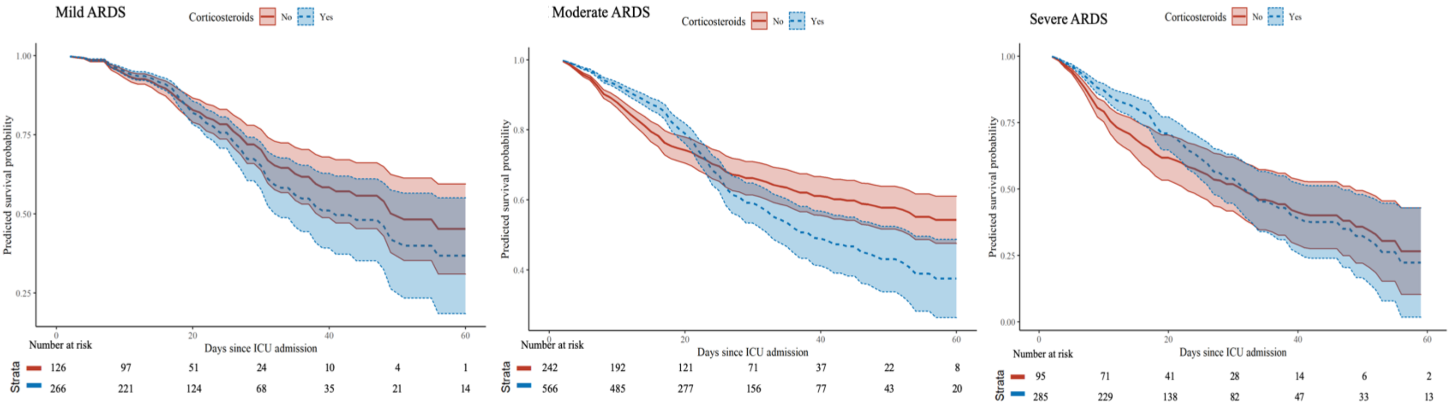
Mild ARDS
Corticosteroids
No
Yes
Predicted survival probability
Days since ICU admission
Number at risk
Strata
126 97 51 24 10 4 1
266 221 124 68 35 21 14
Moderate ARDS
Corticosteroids
No
Yes
Predicted survival probability
Days since ICU admission
Number at risk
Strata
242 192 121 71 37 22 8
566 485 277 156 77 43 20
Severe ARDS
Corticosteroids
No
Yes
Predicted survival probability
Days since ICU admission
Number at risk
Strata
95 71 41 28 14 6 2
285 229 138 82 47 33 13

Figure S7. Post-hoc subgroup sensitivity analysis according with corticosteroid treatment duration. Plot A depicted the survival analysis with cause-specific hazard model for ICU mortality among patients with corticosteroid treatment duration less than seven days compared with none. In a shorter course of treatment, corticosteroids were not associated with short-term effects on survival, but significant negative long-term effects were observed. Plot B showed the survival analysis with cause-specific hazard model for ICU mortality among patients with corticosteroids treatment duration up to seven days or longer. In a longer course of treatment, corticosteroids presented the same time-dependent effect on survival as in the primary analysis.

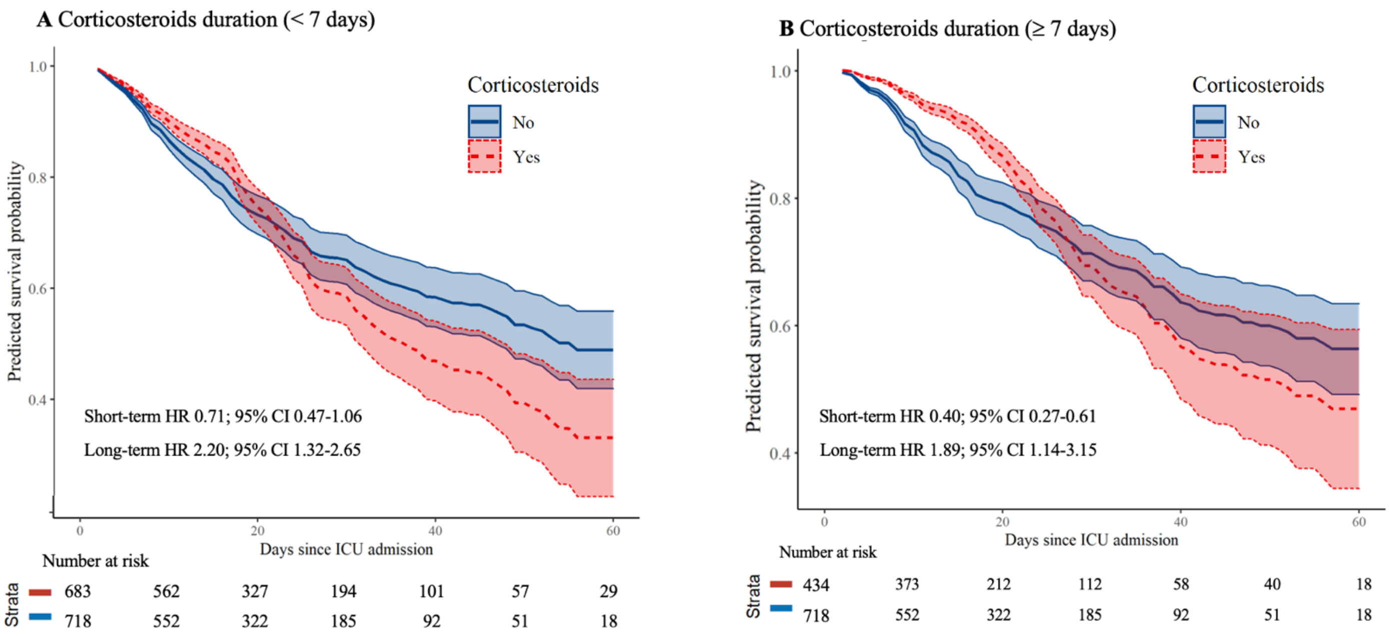

Figure S8. Post-hoc sensitivity subgroup analysis according with tocilizumab (Yes/No).
Exploratory analysis showed that patients who received Corticosteroids plus Tocilizumab had significant association with short-term survival benefit without negative long-terms effects on mortality. In the No Tocilizumab subgroup, corticosteroids had no effects in short-term survival whereas significant negative long-term effects were found.

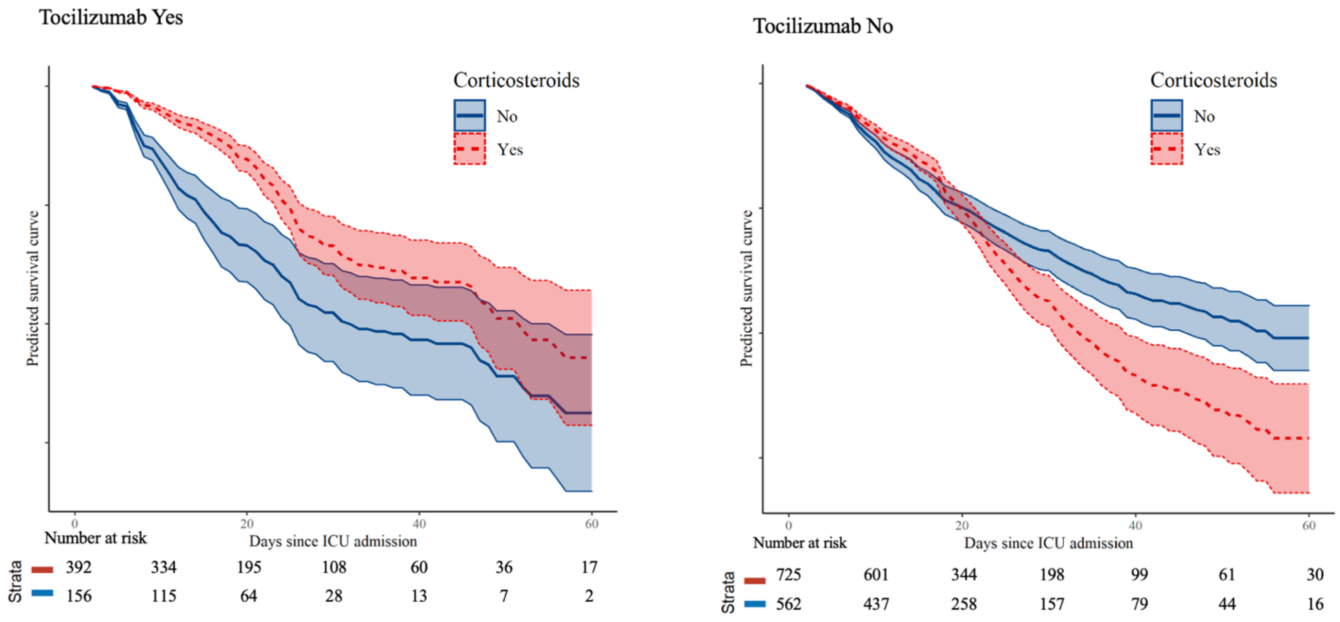

Figure S9. Survival plot of the sub-distribution hazard model for ventilator-free days accounting for competitive event (ICU death) stratified according to corticosteroid treatment. sHR is the sub-distribution hazard ratio expressing the association between corticosteroids and ventilator-free days.

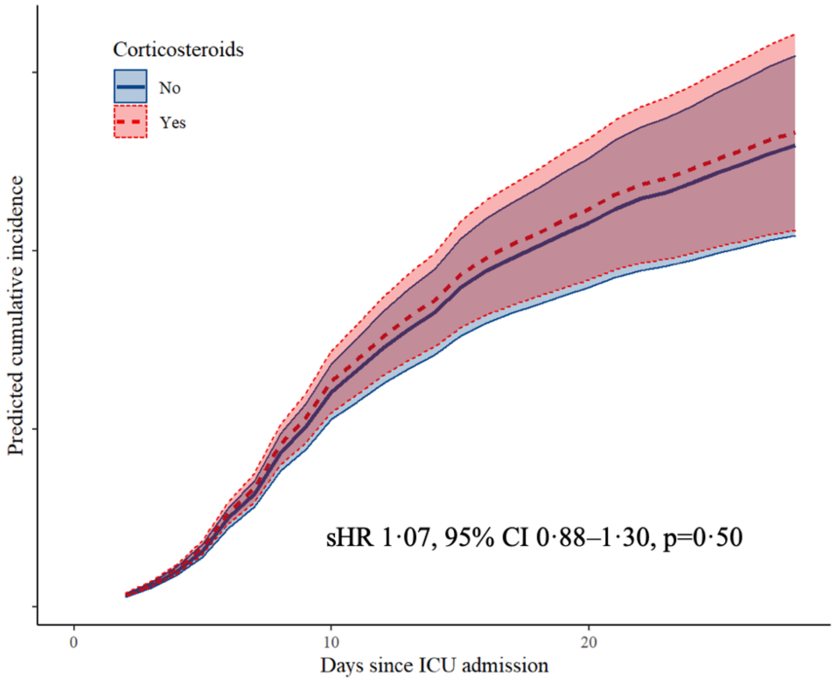

4. COVID-19 SEMICYUC Working group investigators:

Andalucía:
UCI Hospital Universitario Virgen de Valme (Sevilla): Ana Loza; UCI Hospital Quirón (Huelva): Diego Matallana Zapata; UCI Hospital Universitario Puerto Real (Cádiz): Isabel Díaz Torres, Sonia Ibañez Cuadros, María Recuerda Nuñez, Maria Luz Carmona Pérez, Jorge Gómez Ramos, Alba Villares Casas; UCI Hospital Universitario Virgen de la Macarena (Sevilla): María Luisa Cantón, José Javier González Contreras, Helena Pérez Chomón, Nerissa Alvarez Chicote, Alberto Sousa González; UCI Hospital Universitario Reina Sofía (Córdoba): María De Alba Aparicio, Juan Carlos Pozo Laderas; UCI Hospital Universitario de Jerez (Jerez de la Frontera): Angel Estella, Sara moreno Cano. UCI Hospital Infanta Elena (Huelva): Diego Matallana Zapata.
Aragón:
UCI Hospital Nuestra Señora de Gracia (Zaragoza): Ruth Jorge García; UCI Hospital Clínico Universitario Lozano Blesa (Zaragoza): Laura Sánchez Montori, Sandra Herrero García, Paula Abanses Moreno, Carlos Mayordomo García. UCI Hospital General San Jorge(Huesca): Tomás Mallor Bonet, Paula Omedas Bonafonte, Enric Franquesa Gonzalez, Nestor Bueno Vidales, Paula Ocabo Buil, Carlos Serón Arbeloa; UCI Hospital Universitario Miguel Servet(Zaragoza): Isabel Sancho, Pablo Guerrero Ibañez, Pablo Gutierrez, UCI Hospital Obispo Polanco (Teruel): María Concepción Valdovinos, Raquel Canto.UCI Hospital Nuestra Señora de Gracia (Zaragoza): Ruth Jorge García; UCI Hospital Clínico Universitario Lozano Blesa (Zaragoza): Laura Sánchez Montori, Sandra Herrero García, Paula Abanses Moreno, Carlos Mayordomo García. UCI Hospital General San Jorge (Huesca): Tomás Mallor Bonet, Paula Omedas Bonafonte, Enric Franquesa Gonzalez, Nestor Bueno Vidales, Paula Ocabo Buil, Carlos Serón Arbeloa; UCI Hospital Universitario Miguel Servet (Zaragoza): Isabel Sancho, Pablo Guerrero Ibañez, Pablo Gutierrez, UCI Hospital Obispo Polanco (Teruel): María Concepción Valdovinos, Raquel Canto.
Asturias:
UCI Hospital Universitario San Agustín (Avilés): Ana Luz Balán Mariño, María José Gutiérrez Fernández, Marta Martín Cuadrado, Belén García Arias; UCI Hospital Universitario Central de Asturias (Oviedo): Lorena Forcelledo Espina, Lucía Viña Soria, Lorena Martín Iglesias, Lucía López Amor, Elisabet Fernández Rey, Emilio García Prieto. UCI Hospital Cabueñes (Gijón): Débora Fernández Ruíz, Carla Martínez González.
Baleares:
UCI hospital Universitario Son Llatzer (Palma de Mallorca): Lorenzo Socias, Marcio Borges-Sá, María Aranda Pérez, Antonia Socias. UCI Hospital Quirón Salud Palmaplanas (Palma de Mallorca): José Mª Bonell Goytisolo, Inmaculada Alcalde Mayayo, Carlos Corradini, Isabel Ceniceros, Edwin Rodríguez; UCI Hospital Universitario Son Espases (Palma de Mallorca): Jose Ignacio Ayestarán Rota, Mariana Andrea Novo Novo, Joaquim Colomina Climent, Albert Figueras Castilla, Tomàs Leal Rullan, Maria Magdalena Garcias Sastre; UCI Hospital Comarcal d'Inca(Inca): Rossana Pérez Senoff; UCI Hospital Mateu Orfila (Mao): Ramón Fernández-Cid Bouza.
Canarias:
UCI Complejo Hospitalario Universitario Insular—Materno Infantil (Las Palmas de G.C): Juan Carlos Martín González, Carmen Pérez Ortiz, José Luciano Cabrera Santana, Juan José Cáceres Agra, Domingo González Romero, Ana Casamitjana Ortega; UCI Hospital General de la Palma (Tenerife): Luis Alberto Ramos Gómez, Carolina Montelongo Ojeda; UCI Hospital Universitario Dr. Negrín (Las Palmas de G.C): Jordi Solé-Violán.
Cataluña:
UCI Hospital Universitari de Tarragona Joan XXIII (Tarragona): Alejandro Rodríguez, María Bodí, Gerard Moreno, Sandra Trefler, Laura Claverias, Raquel Carbonell, Erika Esteve, Montserrat Olona, Xavier Teixidó. UCI Hospital Universitari Arnau de Vilanova (Lleida): Monserrat Vallverdú Vidal, Begoña Balsera Garrido. UCI Hospital Universitari Vall d'Hebron (Barcelona): Elisabeth Papiol Gallofré, Raquel Albertos Martell, Rosa Alcaráz Peñarrocha, Xavier Nuvials Casals, Ricard Ferrer Roca; UCI Hospital Verge de la Cinta (Tortosa): Eric Adrián Mayor Vázquez, Ferrán Roche Campo, Pablo Concha Martínez, Diego Franch Llasat;UCI Hospital del Mar (Barcelona): Joan Ramón Masclanz, Judith Marín-Corral, Purificación Pérez, Rosana Muñoz, Clara Vila; UCI Hospital Mutua de Terrasa (Terrasa): Francisco Javier González de Molina, Elisabeth Navas Moya, Josep Trenado; UCI Hospital Sant Joan (Reus): Imma Vallverdú, Eric Castañé; UCI Hospital Parc Tauli (Sabadell): Emili Díaz Santos, Gemma Goma, Edgar Moglia.
Cantabria
UCI Hospital Universitario Marqués de Valdecillas(Santander): Borja Suberviola.
Castilla La Mancha:

UCI Hospital Universitario de Guadalajara (Guadalajara): Antonio Albaya Moreno, Carlos Marian Crespo. UCI Hospital Nuestra Señora del Prado (Toledo): Carmen Carolina Sena Pérez, Francisca Arbol Linde.UCI Hospital Universitario de Guadalajara (Guadalajara): Antonio Albaya Moreno, Carlos Marian Crespo. UCI Hospital Nuestra Señora del Prado (Toledo): Carmen Carolina Sena Pérez, Francisca Arbol Linde.

Castilla y León:

UCI Hospital Virgen de la Concha (Zamora): Diana Monge Donaire, Vega Losada Martínez, Nuria Rodrigo Castroviejo, Gerardo Ferrigno, Reyes Beltrán, Carolina Sanmartino, Concepción Tarancón Maján, Alfredo Marcos Gutiérrez; UCI Complejo Asistencial de Segovia(Segovia): Virginia Hidalgo Valverde, Caridad Martín López; UCI Hospital universitario de Burgos (Burgos): Oihane Badallo, María del Valle Ortiz, Rebeca Vara Arlanzón, David Iglesias Posadilla; UCI Hospital Clínica de Salamanca (Salamanca): María Teresa Recio, Juan Carlos Ballesteros; UCI Complejo Asistencial Universitario de Palencia (Palencia).

Ceuta

UCI Hospital Universitario de Ceuta: Enrique Laza Laza.

Extremadura

UCI Hospital San Pedro de Alcántara (Cáceres): Elena Gallego Curto, Mª Carmen Sánchez García; UCI Hospital de Mérida (Mérida): Miguel Díaz-Tavora, Rosa Mancha.

Galicia:

UCI Hospital Montecelo (Pontevedra): Ana Ortega Montes, Isabel Gallego Barbachano, Eva Sanmartín Mantiñán. UCI CHUAC A Coruña (A Coruña): María Lourdes Cordero, Raquel María Rodríguez García, Jorge Gámez Zapata, María Gestal Vázquez. UCI Centro Hospital Universitario de Ferrol (Ferrol): María José Castro Orjales, María Isabel Álvarez Diéguez. UCI Hospitalario Clínico Universitario de Santiago (Santiago de Compostela): Carmen Rivero Velasco, Beatriz Lence Massa; REA CHUAC A Coruña (A Coruña): María Gestal Vázquez.UCI Hospital Lucus Augusti (Lugo): Ignacio Martínez Varela.

Huelva:

UCI Hospital Infanta Elena (Huelva): Diego Matallana Zapata.

Madrid:

UCI IFEMA (Madrid): Alberto Hernández Tejedor; UCI Hospital Príncipe de Asturias (Madrid): Esther Mª López Ramos, Laura Alcázar Sánchez Elvira, Rocío Molina Montero, Mª Consuelo Pintado Delgado, María Trascasa Muñoz de la Peña, Yaiza Betania Ortiz de Zárate Ansotegui, Alejandra Acha Aranda, Juan Higuera Lucas; UCI Hospital de la Princesa (Madrid): Juan Antonio Sanchez Giralt, Marta Chicot Llano, Nuria Arevalillo Fernández, Marta Sánchez Galindo, Ricardo Andino Ruiz, Alfonso Canabal Berlanga; UCI Hospital Clinico San Carlos (Madrid): Miguel Sánchez, Mercedes Nieto; UCI Hospital HLA la Moncloa(Madrid): Eduardo Arias Sarmiento, Adoración Bueno Blázquez, Rosa María de la Casa, Fátima Martín, Samuel González López.

Murcia

UCI Hospital Morales Meseguer (Murcia): Elena Martínez Quintana, Bernardo Gil Rueda, Áurea Higon Cañigral, Laura López Gómez, Pablo Safwat Bayoumi Delis, Augusto Montenegro Muore, Ángel Andrés Agamez Luengas; UCI Hospital Clínico Universitario Virgen de la Arrixaca (Murcia): Enriqueta Andreu Soler, Ana Beatriz Pérez Pérez, José Higinio de Gea García, Rubén Jara Rubio, Silvia Sánchez Cámara, Alba Moreno Flores, José Moya Sánchez, Daniel Francisco Pérez Martínez,-Mª Desamparados del Rey Carrión; UCI Hospital Reina Sofía (Murcia): María José Rico Lledó, Juana María Serrano Navarro, Juan Francisco Martín Ruíz, Julián Triviño Hidalgo, África López Ferrer, Isabel Cremades Navalón; UCI Hospital Santa Lucía (Cartagena): Josefa Murcia Payá, JM Allegre Gallego; UCI Hospital Rafael Méndez (Lorca): María del Carmen Lorente; UCI Hospital Universitario Mar Menor (San Javier):Marta Gonsalvez.

Navarra

UCI Hospital Reina Sofía (Tudela): Ruth González Natera, Raquel Garrido López de Murillo, Tania Ojuel Gros,Raquel Flecha Viguera, Isabel López González; UCI Hospital García Orcoyen(Estella-Lizarra): Adriana García Herrera.

País Vasco

UCI Hospital Universitario de Donostia (Donostia): Loreto Vidaur Tello, Maialen Aseguinolaza, Itziar Eguibar.

Sevilla:

UCI Hospital Universitario Virgen de la Macarena(Sevilla): María Luisa Cantón Bulnes, Jose Javier González Contreras, Helena Pérez Chomón, Nerissa Álvarez Chicote, Alberto Sousa González.

Valencia:

UCI Hospital Universitario de La Ribera (Alzira): Asunción Marqués Parra, Sergio García Marti, Alberto Lorenzo Aguilar, Laura Bellver Bosch, Victor Gascón Sanchez, Sonia De la Guía Ortega. UCI Hospital Dr. Peset (Valencia): Martín Parejo Montell, Alberto Belenguer Muncharaz, Hector Hernández Garces, Victor Ramírez Montero, Mónica Crespo Gómez, Verónica Martí Algarra; UCI Hospital Universitari i Politècnic La Fe (Valencia): Susana Sancho Chinesta, Joaquin Arguedas Cervera, Faustino Álvarez

Cebrian, Begoña Balerdi Pérez, Rosa Jannone Fores, Javier Botella de Maglia; UCI Hospital Clínico Universitario de Valencia (Valencia): Nieves Carbonell Monleón, Jose Ferreres Franco, Ainhoa Serrano Lazaro, Mar Juan Díaz, María Luisa Blasco Cortés; UCI Hospital Virgen de los Lirios de Alcoy (Alicante): Laura Fayos, Julia Giménez, Gaspar Soriano, Ricardo Navarro. UCI Hospital Arnau de Vilanova (Valencia): Sonia Mas, Elena Bisbal, Laura Albert, Johncard Romero, Juan Fernández Cabreara; UCI Hospital Comarcal de Vinarós (Vinarós): Andrea Ortíz.

Principado de Andorra

ICU Hospital Nostra Señyora de Meritxell (Les Esclades): Antonio Margarit Ribas.
